# Participants’ Perspective on the Competence of Mindfulness-Based Interventions Teaching: Development and Validation of the Mindfulness-Based Interventions-Participants’ Assessment of Teaching (MBI:PAT) Questionnaire

**DOI:** 10.1007/s12671-025-02668-8

**Published:** 2025-09-03

**Authors:** Jesus Montero-Marin, Eleanor-Rose Farley, Shannon Maloney, Rebecca Crane, Paul D’Alton, Rebecca Eldridge, Fabio Giommi, Gemma Griffith, Frederick M. Hecht, Verena Hinze, Eric B. Loucks, Clara Strauss, Laura Taylor, Ruth Baer, Willem Kuyken

**Affiliations:** 1 Teaching, Research & Innovation Unit, Parc Sanitari Sant Joan de Déu, Sant Boi de Llobregat, Spain; 2 Department of Psychiatry, University of Oxford, Warneford Hospital, Oxford, OX3 7JX UK; 3 Consortium for Biomedical Research in Epidemiology & Public Health (CIBER Epidemiology and Public Health - CIBERESP), Madrid, Spain; 4 Centre for Mindfulness Research and Practice, School of Psychology and Sports Science, Bangor University, Wales, UK; 5 School of Psychology, University College Dublin, Belfield, Dublin, Ireland; 6 East Coast Mindfulness, Worcester, MA USA; 7 AIM-Italian Association for Mindfulness and Nous-School of Specialization (PsyD) in Psychotherapy, Milan, Italy; 8 Osher Center for Integrative Health, University of California San Francisco, San Francisco, CA USA; 9 Department of Epidemiology, Brown University School of Public Health, Providence, USA; 10 Mindfulness Center at Brown University, Providence, RI USA; 11 School of Psychology, University of Sussex, Brighton, UK; 12 Sussex Mindfulness Centre, Sussex Education Centre, Sussex Partnership NHS Foundation Trust, Mill View Hospital, Hove, UK

**Keywords:** Mindfulness-based, Teaching competence, Participants’ assessment of teaching, MBI:PAT, Questionnaire

## Abstract

**Objectives:**

No participant-rated tool exists for assessing MBI teaching competence. This study aimed to develop and validate the MBI:PAT to address this limitation. The primary objective was to develop a new measure, the Mindfulness-based Interventions: Participants’ Assessment of Teaching" (MBI:PAT), and to evaluate its psychometric properties across several studies using independent samples.

**Method:**

The MBI:PAT was based on a theoretically and empirically supported operational definition of teaching competence and comprised 24 key features across the domains of coverage, pacing, and organization; relational skills; embodying mindfulness; guiding mindfulness practices; conveying course themes through interactive inquiry and didactic teaching; and holding the group’s learning environment. Across five studies, items were generated, refined, and validated using independent samples to assess factor structure, reliability, and validity.

**Results:**

Findings support a 24-item questionnaire, with excellent internal consistency (*ω* = 0.99) and construct, convergent, and divergent validity, with a one-factor structure (CFI = 1.00; TLI = 1.00; RMSEA = 0.04, 90% CI [0.03, 0.05]; SRMR = 0.03). The measure demonstrates robust invariance across age and gender.

**Conclusions:**

The MBI:PAT provides a psychometrically robust measure of MBI teaching competence from participants’ perspective that can be used in teaching, training and research. Future research is needed to explore its performance across a wider range of teaching competence, and its relationship to key process and outcome variables.

**Pre-registration:**

Sub-study (5) was pre-registered (ClinicalTrials.gov: NCT05154266).

**Supplementary Information:**

The online version contains supplementary material available at 10.1007/s12671-025-02668-8.

Mindfulness-based interventions (MBIs) (Kabat-Zinn, 2013; Segal et al., 2018) can enhance mental health, and well-being by helping participants learn mindfulness skills (Feldman & Kuyken, 2019) (Galante et al., 2021; Maloney et al., 2024) Feldman & Kuyken, 2019; Galante et al., 2021; Khoury et al., 2015; Maloney et al., 2024; van Agteren et al., 2021). There is a need to ensure that MBIs are delivered with fidelity to ensure that key mechanisms are targeted and effectiveness, acceptability, and safety are maximized (Crane et al., 2013; Crane & Hecht, 2018; Crane & Kuyken, 2019). As outlined in the UK Medical Research Council (MRC) guidance (Moore et al., 2015), fidelity is a key implementation metric in the context of evaluating complex interventions, yet it continues to be underexplored in behavioral intervention research more broadly (Ginsburg et al., 2021), and in the context of MBI research and practice specifically (Crane & Hecht, 2018; Kechter et al., 2019).

Fidelity addresses different aspects, such as the extent to which the delivered program in fact matches the original curriculum, or the competence of the person teaching the curriculum (Durlak & DuPre, 2008; Tudor et al., 2022). Over time, guidelines for assessing and practicing treatment fidelity in behavioral interventions have been developed and refined, with several frameworks proposed. The Treatment Fidelity Workgroup of the National Institutes of Health Behavior Change Consortium (BCC) is considered the most comprehensive, with its guidelines developed through expert consensus (Bellg et al., 2004). It has consolidated existing definitions, methodologies, and measurement approaches across various frameworks and has introduced new recommendations for ensuring treatment fidelity in behavior change interventions (Bellg et al., 2004). The BCC recommendations detail the following five components of treatment fidelity to be considered when monitoring and reporting efficacy of behavioral interventions: design, training, delivery, receipt, and enactment. (i.) *Design* focuses on methodological strategies to ensure a study can effectively test hypotheses and deliver the intervention consistently across conditions, with plans for handling implementation challenges; (ii.) *Training* ensures that interventionists are properly equipped to deliver the intervention to the target population, using standardized curriculum manuals to acquire and maintain provider skills while accounting for differences; (iii.) *Delivery* involves monitoring the intervention to ensure it is implemented as intended, controlling for variations in treatment delivery and adherence to protocol; (iv.) *Receipt* emphasizes strategies to enhance and monitor participants' understanding and application of intervention skills during the intervention, ensuring comprehension and performance; and (v.) *Enactment* ensures that participants apply the learned skills in their daily lives outside the intervention setting. It is usually considered that design, training, and delivery components focus on the treatment provider while receipt and enactment components focus on the participant (Kechter et al., 2019).

The Treatment Fidelity Tool for MBIs (Kechter et al., 2019) is an adapted framework based on the BCC guidelines, designed to assist researchers in monitoring and reporting fidelity in a clear and standardized manner. This tool offers researchers an opportunity to enhance the transparency and interpretability of the evidence base for MBIs. However, this tool, in turn, needs to rely on additional instruments that offer specific measures for the characteristics of the five components mentioned above. For example, there are different assessment instruments to evaluate delivery (i.e., the extent to which the intervention is delivered as intended) relying on expert ratings of MBI teaching. Some examples are the Mindfulness-Based Interventions: Teaching Assessment Criteria (MBI:TAC) (Crane et al., 2013), the Mindfulness-Based Relapse Prevention-Adherence and Competence Scale (MBRP-AC) (Chawla et al., 2010), and the Mindfulness-Based Cognitive Therapy-Adherence Scale (MBCT-AS) (Segal et al., 2002). Whilst they all assess curriculum adherence, two additionally assess teaching competence (MBI: TAC, and MBRP-AC). The MBI:TAC (see Online Resource 1) is the most widely used, because it assesses curriculum adherence and teaching competence in both research and teacher training contexts (Crane & Kuyken, 2019). It comprises the following six domains: coverage, pacing, and organization of session curriculum; relational skills; embodying mindfulness; guiding mindfulness practices; conveying course themes through interactive and didactic teaching; and holding the group-learning environment. Each domain is captured by several key features. MBI teaching is rated by expert observers on each of the six domains on a six-point scale, ranging from “incompetent” to “advanced,” and raters also make an overall rating. The MBI:TAC development involved extensive work to establish its internal consistency and face validity (Crane et al., 2012), and subsequent work has shown that with adequate training, assessments can be performed with a good inter-rater reliability (overall intraclass correlation coefficient = 0.81) (Crane & Kuyken, 2019). However, its relationship to outcomes has not yet been demonstrated (Huijbers et al., 2017), although research suggests that teaching competence, as measured by a version of the MBI:TAC adapted to school settings, might be related to participants’ interests and attitudes towards the program (i.e., responsiveness), as well as to their degree of engagement in terms of home-based mindfulness practice (Montero-Marin et al., 2023).

All existing formal MBI teaching assessment tools to evaluate delivery rely on expert ratings and there is currently no formal psychometrically evaluated measure of MBI teaching from the participants’ perspective. While participants cannot know the intended MBI curriculum, they can provide a valuable perspective on teaching competence as they experience it. Participants’ perspectives can provide direct feedback to teachers, as well as contribute to quality control, learning, and teaching improvements. In addition, an approach focused on participants' perspectives may be less expensive and time consuming, and more feasible to implement in many settings, as it does not require video recordings of sessions and the need of expert evaluators. This is important as costs and time constraints can be barriers to the assessment of fidelity, which can be onerous in research and clinical settings using complex interventions (Ginsburg et al., 2021). Finally, an approach focused on participants' perspectives could facilitate MBI research, ultimately enhancing MBI effectiveness (Monteiro, 2020).

The studies outlined in the current paper set out to develop a measure of teaching competence that had high accessibility in terms of framing, brevity, and utility (i.e., short and easy to use for diverse populations); mapped onto established best practices in MBI teaching assessment (the MBI:TAC); and is suitable for both practice and research. The development and evaluation of the MBI:PAT involved five sub-studies: (1) generated, reviewed, and refined items; (2) reduced items and conducted exploratory analyses; (3) confirmed the factor structure by psychometric cross-validation; (4) assessed data clustering, invariance, and floor/ceiling effects; and (5) evaluated convergent/discriminant validity. This involved a scientific advisory group, consisting of 20 MBI researchers and instructors from several countries (Italy, Netherlands, UK, US, and Spain), and pilot and field testing across several centers delivering MBIs.

The development of a formal and psychometrically robust participant perspective on MBI teaching supports the integrity of MBI teaching and training, quality assuring MBI teaching, and provides a tool for addressing research questions about MBI acceptability, effectiveness, cost effectiveness and implementation that require measures of MBI fidelity and participants’ perspectives on MBI teaching.

## Study 1

The aim of Study 1 was to develop and refine a questionnaire based on the MBI:TAC, designed to be completed by participants of an MBI, and to be accessible and applicable to a wide range of MBI formats and programs.

### Method

#### Participants

An intentional sampling strategy was employed to leave an adequate pool of potential participants for larger-scale field testing, reducing the risk of participant exposure to early versions of the MBI:PAT, which could affect their responses and limit the usefulness of their data. Study 1 involved several groups of participants: (i.) *Focus Groups and Interview*: The first two focus groups were conducted with participants based in the UK (*n* = 10), with an age range between 40 and 80 years, and 60.00% identified as female. The third focus group was conducted with participants from the US (*n* = 8), with 66.70% females, aged between 25 to 60 years. Additionally, a young person (UK male, aged 13 years) with no prior MBI experience was consulted and interviewed to ensure the language was accessible to a younger audience; and (ii.) *Pilot Testing*: Twenty participants who attended MBI classes were involved in the pilot testing phase of the initial MBI: PAT. These participants were recruited via email from participating mindfulness centers (Bangor University Centre for Mindfulness Research and Practice, Brown University Mindfulness Center, Radboud University Medical Center for Mindfulness, Nottingham Mindfulness Centre, Sussex Mindfulness Centre, University College Dublin, and the Oxford Mindfulness Foundation).

#### Procedure

The advisory group first assessed whether participants could rate each MBI:TAC domain and key feature, identifying issues with language complexity and length. Three iterative focus groups were held to develop and refine the MBI:PAT items, ensuring clarity and accessibility for diverse users, including young people. Feedback from these groups and a young novice led to further item revisions. The scientific advisory group and additional experts reviewed the final item pool for alignment with the MBI:TAC. Standardized instructions were created, and the refined items were prepared for pilot testing, where participants provided feedback on length, content, and clarity through an anonymous online survey.

#### Measures

The MBI:PAT items were developed to map onto the relevant MBI:TAC domains and key features. The item pool initially consisted of 87 items, covering each MBI:PAT domain and key feature with at least one item. The items were created based on language and phrasing suggested during the focus groups and were further refined to ensure they were accessible to a wide range of populations, including young people. During pilot testing, participants were asked whether they did not understand or could not answer the items. In those cases, they were asked to explain what it was about the item that made it unanswerable and provide suggested improvements. They were also asked for general comments on the questionnaire in terms of length, content, and any other suggested improvements. The final items used a Likert response scale ranging from 1 (*Not at all*) to 6 (*Outstanding*) to map onto the MBI: TAC rating scale.

#### Data Analyses

The advisory group first reviewed each of the MBI:TAC domains and key features to establish by means of consensus if participants could rate each domain and key feature. Then, we began with three focus groups, comprising people who had attended an MBI in either the UK or US. The purpose of these focus groups was to co-create the MBI:PAT with people who had previously participated in MBIs, and to ensure language suitability and item comprehension. Focus groups were co-led by a researcher with experience in public engagement and an MBI teacher. All groups were conducted online, using videoconferencing. The interviews were audio recorded, with opt-in verbal permission provided by all participants, and the recordings were transcribed verbatim. The focus groups were conducted in an iterative manner, with the insights obtained in previous groups informing the subsequent focus group. During the pilot testing phase, participants’ feedback on the initial version of the MBI:PAT was collected via an anonymous online survey. The data included responses to the items, distribution of responses (to identify potential floor or ceiling effects), and ratings of item acceptability. Participants’ suggestions for item improvement were also collected and considered.

### Results

The advisory group's initial review of the MBI:TAC domains revealed several challenges that needed to be addressed for the successful development of the MBI:PAT. The following issues were identified: (i.) the language required specialized knowledge of mindfulness and MBI teaching, underscoring the need for alterations to make it more accessible; (ii.) with six domains, consisting of at least four key features each, the MBI:TAC was considered too extensive to be used effectively as a self-reported questionnaire for participants, pointing to the need to aim to have no more than one item per key feature; and (iii.) participants may struggle to provide a rating on some MBI:TAC domains and key features. For example, Domain 1 (coverage, pacing, and organization of session curriculum) includes teaching elements such as curriculum adherence, which participants would have no knowledge of and therefore would be unable to comment on. However, participants could comment on other elements such as level of organization of the MBI course by the teacher, room set-up and materials, flow of the course, etc. For Domain 3 (embodying mindfulness), the MBI:TAC uses descriptive language of the MBI teacher’s embodying that requires expert knowledge. For Domain 4 (guiding mindfulness practices), Key Features 2 (i.e., key learning for each practice) and 3 (i.e., particular elements for each practice) refer to specific practices and activities that are used only in some MBIs (Crane & Kuyken, 2019). Finally, the MBI:PAT was intended to map onto different MBI formats, so items needed to be applicable for a wide range of MBI programs and adaptations. Taking all these expert considerations into account, MBI:PAT items were generated and refined based on focus group work.

MBI:PAT item generation involved developing an item pool (ranging from two to three times the number of key features, to allow for item choice, and from which to select the best items) that mapped onto the relevant MBI:TAC domains and key features. The first focus group was open and broad, each domain and key feature was explained in turn by the focus group leaders, and participants were asked about the language and the specific words they would use to describe the quality of MBI teaching they received. The key phrases and words that were most easily understandable were then put forward to the next focus group. The second focus group consisted of different people and repeated and extended this process by refining and elaborating on the work of the first group. Participants were asked if they felt the words and key phrases were understandable, and they discussed what they thought they understood and whether the question could be improved in terms of phrasing. The dataset of words and phrases generated from the second group was used to write a first tentative set of items. A third focus group was then conducted to review and suggest refinements to the first item pool. Participants of the third focus group were asked whether they understood the items, if they could be improved, and if the items mapped onto the respective domains and key features. This focus group was held in the US to ensure items mapped across British and American English. Because participants in the third focus group largely endorsed the item pool, we progressed to the next step. This step was to ensure that the language used in the MBI is accessible to a range of populations, including young people. To that end, we consulted a young person with no prior MBI experience. The input provided by the young person led to revisions in the wording of a few items.

The item pool was reviewed against the MBI:TAC domains and key features by the scientific advisory group (along with additional MBI researchers and teachers, with an in-depth understanding of the MBI:TAC), ensuring the items had not deviated too far from the MBI:TAC. We then developed standardized instructions for the MBI:PAT, and additional instructions for the pilot testing, which explained to participants that the MBI:PAT was piloted and therefore, their mindfulness teachers would be unaware of their ratings. In total, 87 items were generated, some of which were specific for pilot testing only to help improve the measure. Each MBI:TAC domain and underpinning key feature was covered by at least one item, and most contained several items.

In the pilot testing phase, participants’ distribution of responses (showing potential floor or ceiling effects) as well as ratings of acceptability (including suggestions for improvement) determined which items needed to be revised or deleted by the scientific advisory group. All of this led to the creation of a refined version of 73 items. These items were prepared for larger-scale field testing. See Online Resource 2 to view the 73 items created in Study 1 for larger scale field testing.

### Discussion

Based on the MBI:TAC, the MBI:PAT is designed to assess participants' perceptions of the quality of MBI teaching, with an emphasis on simplifying the language used and ensuring comprehensibility for diverse populations, including those with no prior knowledge of mindfulness practice. The challenges identified by the advisory group's initial review of the MBI:TAC included the need for accessible language, a manageable number of items, and the ability to provide meaningful ratings on the MBI:TAC domains (Crane et al., 2013). The focus groups process played a critical role in refining the item pool, ensuring that the language was comprehensible and that the items were appropriate for the target population. The iterative nature of the focus groups allowed for continuous improvement of the items, leading to a more robust and user-friendly questionnaire. Based on the feedback of the focus groups and interview, the scientific advisory group developed and refined an initial 87-item pool. The pilot testing phase confirmed the acceptability of the items and provided valuable insights into item discrimination, which guided the refinement process. The resulting 73-item version of the MBI:PAT (see Online Resource 2), grounded in both expert input and participant feedback, was well-positioned for larger-scale field testing.

## Study 2

Based on the refined item pool, Study 2 aimed to reduce the MBI:PAT item pool and start to explore its validity (in terms of factor structure) and reliability (in terms of internal consistency). To do this, we followed the theoretically supported MBI:TAC structure (Crane et al., 2013) that outlines 24 key features of teaching competence that may be applied to the participants’ perspective within the six core domains. A summary of the structure of the MBI:PAT in terms of main domains and key features is provided in Online Resource 2.

### Method

#### Participants

The characteristics of Stage 2 study participants (i.e., calibration sample) are summarized in Table 1. Participants (*n* = 201) had a mean age of 45.60 years (*SD* = 11.60), were primarily female (62.70%), married (49.70%), with no dependents (42.30%), and had occupations mostly in the management (35.30%) and services (28.10%) sectors. This sample size was considered adequate for our exploratory analysis to have enough observations per variable (i.e., between 5 and 10 participants per item) to obtain robust results (Hair, 2009). Participants had received a median of eight (interquartile range: [8, 8]) mindfulness sessions prior to completing the survey. The most common MBIs were Mindfulness-Based Cognitive Therapy for Life (MBCT-L; 86.10%) (Feldman & Kuyken, 2019; Strauss et al., 2021) and Mindfulness-Based Stress Reduction (MBSR; 4.50%) (Kabat-Zinn, 2013). The MBIs were most frequently delivered by the Oxford Mindfulness Foundation (91.00%). Most of these MBIs were delivered synchronously online (98.50%); the remainder were face-to-face. A total of 27 MBI teachers taught the interventions. They had completed a training program, received an official certification attesting to their competence, and met the good practice criteria set out by the British Association of Mindfulness-Based Approaches (BAMBA; https://bamba.org.uk/).

**Table 1:** Characteristics of the study participants

	Calibration sample (*n* = 201)	Validation sample (*n* = 199)	Total (*n* = 400)
Age (in years): *M* (*SD*)	45.55 (11.58)	47.78 (12.65)	46.52 (12.09)
Gender (women): *n* (%)	126 (62.70)	88 (44.20)	88 (53.30)
Marital status: *n* (%)			
* Never married*	52 (31.10)	22 (36.70)	74 (32.60)
* Married*	83 (49.70)	29 (48.30)	110 (49.30)
* Separated/divorced*	17 (10.20)	5 (8.30)	12 (9.70)
* Widowed*	3 (1.80)	1 (1.70)	4 (1.80)
Dependents: *n* (%)			
* 0*	85 (42.30)	42 (21.10)	127 (31.80)
* 1*	42 (20.90)	7 (3.50)	49 (12.30)
* 2*	24 (11.90)	7 (3.50)	31 (7.80)
* 3*	7 (3.50)	3 (1.50)	10 (2.50)
* 4 or more*	1 (0.50)	0 (0.00)	1 (0.30)
Occupation: n (%)			
* Management*	59 (35.30)	27 (21.30)	86 (30.90)
* Services*	47 (28.10)	47 (37.00)	94 (33.80)
* Government*	8 (4.80)	1 (0.80)	9 (3.20)
* Retired*	11 (6.60)	10 (7.90)	21 (7.60)
* Student*	5 (3.00)	9 (7.10)	14 (5.00)
* Unemployed*	4 (2.40)	3 (2.40)	7 (2.50)
* Other*	20 (12.00)	27 (21.30)	47 (16.90)
Yearly income: *n* (%)			
* Less than £10,000*	14 (7.00)	6 (3.00)	20 (5.00)
* £10,000 to £25,000*	24 (11.90)	10 (5.00)	34 (8.50)
* £25,000 to £50,000*	36 (17.90)	15 (7.50)	51 (12.80)
* £50,000 to £75,000*	26 (12.90)	8 (4.00)	34 (8.50)
* £75,000 and above*	33 (16.40)	10 (5.00)	43(10.80)
Total MBI sessions received: Md (IQR)	8 (8, 8)	8 (8, 11)	8 (8, 9)
Type of MBI course received: *n* (%)			
* MBCT*	1 (0.50)	0 (0.00)	1 (0.30)
* MBCT-L*	173 (86.10)	129 (64.80)	302 (75.50)
* MBCT-TIF*	6 (3.00)	70 (35.20)	76 (19.00)
* MBSR*	9 (4.50)	0 (0.00)	9 (2.30)
* Other*	3 (1.50)	0 (0.00)	3 (0.80)
Centre of MBI delivery: *n* (%)			
* OMC*	183 (91.00)	199 (100.00)	382 (95.50)
* Other*	11 (5.50)	0 (0.00)	11 (3.10)

*M* mean, *SD* standard deviation, *Md* median, IQR interquartile range, *n *frequencies, % percentages, *MBI* Mindfulness-Based Program, *OMC* Oxford Mindfulness Centre. “Prefer not to say” was an allowed option in all the sociodemographic data survey. Consequently, in the calibration sample, a total of 33 participants did not report on age, 37 participants did not report on gender, 45 participants did not report on marital status, 42 participants did not report on dependents, 47 participants did not report on occupation, 68 participants did not report on yearly income, 9 participants did not report on the type of MBI course received, 7 participants did not report the center. In the validation sample, a total of 70 participants did not report on age, 69 participants did not report on gender, 142 participants did not report on marital status, 140 participants did not report on dependents, 75 participants did not report on occupation, 150 participants did not report on yearly income

#### Procedure

Participants were recruited using an existing database of individuals, who consented to be contacted and had completed a mindfulness course at the Oxford Mindfulness Foundation. Additionally, participants were recruited through direct approaches in other mindfulness centers around the world (Centre for Mindfulness Research and Practice at Bangor University and East Coast Mindfulness), which shared the link with participants, who both consented to be contacted and had completed a mindfulness-based course. Participants completed an anonymous online survey in Qualtrics (www.qualtrics.com) between May-2021 and September-2021.

#### Measures

The survey contained the total pool of MBI:PAT items that were generated, reviewed, and refined within Study 1 (see Online Resource 2), as well as basic sociodemographic questions. The MBI:PAT item pool consisted of the 73 items from Study 1, across six domains comprising 24 key features (see Online Resource 2). Items were presented in a random order across the six main domains to minimize the impact of completion fatigue on our outcomes. Participants were instructed to indicate how true each statement was, using a 6-point Likert scale ranging from 1 (*Not at all*) to 6 (*Outstanding*).

#### Data Analyses

To establish the MBI-PAT items ability to differentiate across the range of teaching quality, we first describe the distributions of items generated in Stage 1, using means (*SD*s), skewness, kurtosis, and medians. To establish items unique contribution and redundancy the polychoric correlation matrix was also calculated. To reduce the pool of items and create a scale of manageable length for use in a variety of contexts, we selected one item per key feature, using the highest corrected item-total (i.e., item-rest) correlation within each MBI:PAT key feature. This helped ensure theoretical representativeness of selected items within the MBI:PAT framework. We then explored the psychometric characteristics of the selected items by determining the underlying factorial structure; closeness to one-dimensionality; relationships and patterns among the items to identify potential latent factors; and internal consistency.

To determine the underlying factorial structure, we used both Schwartz’s Bayesian information criterion (BIC) and Parallel Analysis (PA) as dimensionality tests. Parallel analysis was based on optimal implementation and minimum rank factor analysis. The number of random correlation matrices was 500, which were generated from the permutation of sample values. We considered both the 95th percentile and the mean of the random percentage of variance. We assessed unidimensionality using three indices: Unidimensional Congruence (UniCo), Explained Common Variance (ECV), and the Mean of Item Residual Absolute Loadings (MIREAL). The UniCo is the congruence between the actual loading matrix and the loading matrix that would be obtained if the unidimensional model is true. The closer the UniCo value is to 1 (cut-off value > 0.95) the more strongly the actual loading matrix represents the unidimensional loading matrix (Ferrando & Lorenzo-Seva, 2019). MIREAL represents the absolute loadings on the second factor (using minimum rank factor analysis), as a measure of the departure from one-dimensionality at the item level. Values of MIREAL lower than 0.30 indicate no substantial bias if a unidimensional solution is fitted (Grice, 2001). ECV measures the proportion of common variance attributable to the first canonical factor (i.e., the factor that explains most common variance),and represents the dominance of the first factor over the other factors. It is proposed that the ECV value should be > 0.85, if a solution is to be accepted as essentially unidimensional (Rodriguez et al., 2016).

The structure of the underlying relationships and patterns among the items of the MBI:PAT was assessed using exploratory factor analysis (EFA) with the weighted least squares mean- and variance-adjusted estimator (WLSMV) for ordinal variables. Given our primary interest in participant-level effects, we adopted a design-based approach. Thus, we specified an overall model for the complex data to infer findings to the lower-level sampling units after controlling for the potential teacher-level (cluster) effects (Wu et al., 2017). For that, we adjusted the standard errors of parameter estimates to accommodate the group-level sampling design, by using the cluster-robust standard error algorithm (sandwich estimator). An evaluation of the magnitude of any teacher-level effects, as well as a comparison of factor loadings from the two-level model and the design-based approach, is provided in Stage 5. The following goodness-of-fit indices were examined with their respective cut-off points (Schermelleh-Engel et al., 2003): the comparative fit index (CFI; ≥ 0.95 for good, ≥ 0.90 for acceptable); the Tucker–Lewis index (TLI; ≥ 0.95 for good, ≥ 0.90 for acceptable); the root-mean-square error of approximation (RMSEA; ≤ 0.06 for good, ≤ 0.10 for acceptable) with its 90% confidence interval (90% CI); and the standardized root mean square residual (SRMR; ≤ 0.06 for good, ≤ 0.10 for acceptable). Furthermore, we calculated the standardized factor loadings (*λ*); the uniqueness terms (*δ*) (i.e., a measure of unexplained variance in observed variables); and the average variance extracted (AVE) (i.e., the proportion of variance in the observed variables that is explained by the latent construct in relation to the variance attributed to measurement error).

Internal consistency was determined by calculating the McDonald’s omega (*ω*) index, with coefficients ≥ 0.70 indicating adequate internal consistency values (DeVellis & Thorpe, 2021). McDonald’s *ω* has the advantage of considering the strength of the association between items and constructs, as well as the item-specific measurement errors and, therefore, provides a realistic estimate of true reliability (McDonald, 2013). We also calculated the construct replicability (*H*), which indicates how well latent variables are defined by evaluating the proportion of the factor variance that can be accounted for by its indicators, with *H* ≥ 0.70 indicating a reasonable definition and *H* ≥ 0.80 suggesting an adequately defined construct (Hancock & Mueller, 2001). Mplus v8.10 and Factor v12.03.02 statistical packages were used for the analyses.

### Results

Tc 3. The mean item values were above 5 (using a range from 1 to 6) in almost all cases, suggesting a skew towards positive participant ratings of MBI teaching competence and lack of differentiation teaching quality.

The polychoric correlations of the initial pool of items are provided in Online Resource 4. The mean inter-item polychoric correlation of the initial pool of items was *r* = 0.69, meeting commonly recognized benchmarks for strong internal consistency (Clark & Watson, 1995). All these items showed high (within-domain and between-domain) item-rest correlations, and all these correlations were in the same direction (see Online Resource 5). To reduce the pool of items selecting the most consistent ones, we chose one item per key feature of each domain, using the highest corrected item-rest correlation within each of the MBI:PAT key features. The item-rest correlation values for the selected items ranged from 0.64 (Item 14: “The pace of the classes was right for me (for example, activities were not too rushed or too slow)”), to 0.89 (Item 37: “The teacher responded calmly to challenging situations”). An exception was made for Domain 6 (Holding the group learning environment), Key Feature 22 (Group development – clear management of group development processes over the eight weeks, particularly the management of beginnings, endings, and challenges within the group). Although the Item 68 (“The teacher dealt skillfully with challenging group members (for example, people who argued with the teacher or those who talked too much)”) presented the highest item-rest value (0.78), it was established by consensus that Item 66 (“The teacher ensured the group kept to a set of “ground rules” throughout the classes”), with an item-rest value of 0.76, would better represent this key feature, in keeping with the MBI:PAT theoretical framework. Online Resource 5 presents the item selection process in detail. The final list of 24 items is shown in Online Resource 6.

Using the final list of 24 items (each corresponding to a key feature), results from both the BIC and PA dimensionality tests suggested a one-factor structure (see Online Resource 7). This solution explained a high percentage (65.70%) of total variance. The closeness to one-dimensionality of the selected group of items was supported, with values of UniCo = 0.99, ECV = 0.95, and MIREAL = 0.15. Online Resource 8 shows the polychoric correlation matrix for the 24 selected items. The mean inter-item polychoric correlation for the 24 selected items was high (*r* = 0.74). The goodness of fit for the one-factor model was good (CFI = 0.99, TLI = 0.99, RMSEA = 0.06, 90% CI [0.06, 0.07], SRMR = 0.04). The standardized factor loadings for the one-factor model were higher than 0.72 in all cases, and the uniqueness terms were lower than 0.48 (Table 2). Finally, the AVE value was 0.73 and the internal consistency of the factor as well as its construct replicability were high (with both *ω* and *H* showing a value of 0.99).

**Table 2:** Descriptive data and factor loadings of the pool of selected items

Calibration sample (*n* = 201)								
Item	*M*	*SD*	Skew	Kurt	Mn	*r*_tot_	*λ*	*δ*
4	5.12	0.79	−0.89	1.10	5.00	0.69	0.77	0.41
7	4.94	1.13	−1.35	1.93	5.00	0.68	0.75	0.44
9	5.13	0.94	−1.03	0.90	5.00	0.68	0.77	0.41
12	5.48	0.70	−1.42	2.68	6.00	0.77	0.85	0.28
14	5.16	0.95	−1.23	1.81	5.00	0.63	0.73	0.47
15	5.59	0.78	−2.49	8.02	6.00	0.80	0.90	0.19
17	5.33	0.89	−1.86	5.21	6.00	0.84	0.90	0.19
23	5.30	0.90	−1.48	2.78	6.00	0.84	0.91	0.17
26	5.44	0.87	−2.06	5.60	6.00	0.81	0.89	0.21
28	5.26	0.89	−1.52	3.28	5.00	0.75	0.82	0.33
34	5.56	0.67	−1.53	2.18	6.00	0.80	0.92	0.15
36	5.38	0.74	−1.05	0.65	6.00	0.88	0.94	0.12
37	5.37	0.78	−1.34	2.06	6.00	0.86	0.94	0.12
40	5.51	0.80	−2.27	7.13	6.00	0.81	0.89	0.21
42	5.44	0.75	−1.78	5.83	6.00	0.75	0.85	0.28
46	5.47	0.70	−1.22	1.11	6.00	0.79	0.88	0.23
54	5.41	0.80	−1.41	1.95	6.00	0.79	0.90	0.19
55	5.28	0.90	−1.63	3.74	5.00	0.82	0.91	0.17
59	5.21	0.91	−1.52	3.58	5.00	0.89	0.94	0.12
61	5.47	0.74	−1.53	2.83	6.00	0.77	0.87	0.24
64	5.38	0.84	−1.68	4.06	6.00	0.82	0.89	0.21
66	5.31	0.78	−1.12	1.41	5.00	0.70	0.81	0.34
69	5.26	0.86	−1.31	1.79	5.00	0.87	0.94	0.12
72	5.32	0.87	−1.85	4.91	5.00	0.85	0.91	0.17
Validation sample (*n* = 199)								
Item	***M***	***SD***	**Skew**	**Kurt**	**Mn**	***r***_**tot**_	**λ**	**δ**
4	5.21	0.80	−1.11	1.78	5.00	0.71	0.75	0.44
7	5.05	1.09	−1.44	1.93	5.00	0.68	0.77	0.41
9	5.19	1.00	−1.55	2.60	5.00	0.78	0.83	0.31
12	5.56	0.62	−1.37	1.87	6.00	0.70	0.83	0.31
14	5.18	0.96	−1.44	2.27	5.00	0.71	0.80	0.36
15	5.56	0.76	−1.98	4.05	6.00	0.82	0.90	0.19
17	5.34	0.91	−1.85	4.16	6.00	0.87	0.92	0.15
23	5.32	0.98	−1.81	3.54	6.00	0.85	0.90	0.19
26	5.48	0.77	−1.60	2.58	6.00	0.83	0.90	0.19
28	5.29	0.90	−1.74	4.07	5.00	0.86	0.90	0.19
34	5.52	0.76	−2.27	7.70	6.00	0.81	0.92	0.15
36	5.44	0.75	−1.49	2.65	6.00	0.88	0.94	0.12
37	5.47	0.70	−1.39	2.04	6.00	0.85	0.93	0.14
40	5.49	0.76	−1.58	2.17	6.00	0.82	0.89	0.21
42	5.41	0.79	−1.49	2.40	6.00	0.82	0.90	0.19
46	5.43	0.75	−1.38	2.12	6.00	0.85	0.92	0.15
54	5.31	0.89	−1.57	3.37	6.00	0.80	0.87	0.24
55	5.27	0.91	−1.54	2.99	5.00	0.82	0.88	0.23
59	5.28	0.95	−1.78	3.70	5.00	0.92	0.95	0.10
61	5.53	0.66	−1.17	0.61	6.00	0.80	0.92	0.15
64	5.38	0.87	−1.68	3.00	6.00	0.86	0.91	0.17
66	5.19	0.90	−1.43	2.50	5.00	0.70	0.80	0.36
69	5.31	0.81	−1.48	3.16	5.00	0.82	0.88	0.23
72	5.34	0.88	−1.66	3.09	6.00	0.88	0.93	0.14

*M* mean, *SD* standard deviation, *Skew* skewness, *Kurt* kurtosis, *Mn* median, *r*_*tot*_ item-rest correlation, *λ* factor loadings, *δ* uniqueness terms. The original numeration of the items is maintained according to the order in the total group. In terms of teachers (clusters), a total of *n* = 10 participants in the calibration sub-sample answered that they were rating “other teacher, not listed” and a total of *n* = 9 participants in the calibration sub-sample responded that “prefer not to say” the name of the teacher. These two types of response were used to group participants into two different clusters that were added for the analysis (all the participants in the validation sample fulfilled the name of the teacher being rated). Discarding these participants in the analysis produced small variations on item intra-class correlation coefficient (ICC) values

### Discussion

Together, the results from Study 2 indicate that the 24 selected items for the MBI:PAT are adequately related to each other, consistently measuring the underlying construct of MBI teaching competence from the participant’s perspective. We have observed that a significant portion of the variance in the items was explained by a single latent construct, which, in turn, accounted for a high percentage of the factor variance through its indicator items. This suggests that the final set of 24 items for the MBI:PAT (each covering a key feature) may represent a reliable and coherent measure of participants' assessments of MBI delivery in terms of teaching competence (Durlak & DuPre, 2008; Tudor et al., 2022), using a general factor that integrates all key features of MBI teaching competence, as proposed in Study 1.

## Study 3

Using the 24-item MBI-PAT developed in Study 2, Study 3 set out to confirm the factor structure of the MBI:PAT and the psychometric properties that emerged in Study 2 in a new validation sample of MBI participants. To do this, we used the final set of 24 items and conducted confirmatory factor analyses (CFA) to evaluate the one-dimensional structure observed in Study 2 and evaluated its internal consistency. We hypothesized that the theoretically and empirically derived one-dimensional structure identified in Study 2, would be a good fit to the data for the MBI:PAT, with adequate internal consistency.

### Method

#### Participants

The participant characteristics for Study 3 (i.e., cross-validation sample) are shown in Table 1. Participants (*n* = 199) had a mean age of 47.80 (*SD* = 12.70) years; 44.20% were female, 48.30% married, 21.10% with no dependents, 37.00% had occupations in the services sector (e.g., health, education), and 21.30% in the management sector, and they had received a median of 8 (interquartile range [8, 11]) mindfulness sessions. We deemed this sample size sufficient for our confirmatory objective, following the general guideline of ensuring an adequate number of observations per variable (typically between 5 and 10 participants per item). This ensures the generation of robust and stable findings in our confirmatory factor analysis. The mindfulness interventions that were most frequently received were MBCT-L (64.80%), and Mindfulness-Based Cognitive Therapy–Taking it Further (MBCT-TiF; 35.20%), which is an advanced curriculum for graduates of MBIs (Maloney et al., 2024). All mindfulness interventions were delivered synchronously online and were delivered by the Oxford Mindfulness Foundation. A total of 18 certified MBI teachers, who met good practice criteria as set out by the BAMBA (https://bamba.org.uk/), were involved in teaching mindfulness interventions and were rated by participants.

#### Procedure

Participants were recruited using an existing database of individuals who consented to be contacted and had already completed a mindfulness course at the Oxford Mindfulness Foundation. To complete the ratings, participants were directed to an anonymous online survey in Qualtrics (www.qualtrics.com) between August-2022 and December-2022. The survey outlined the main objective (i.e., to test a measurement tool to assess MBI teaching competence). The participation was voluntary in that participants could exit the survey link at any time without consequences.

#### Measures

The survey contained the total pool of MBI:PAT items that were generated, reviewed, and refined within Study 1, but only the final subset of selected 24 MBI:PAT items were analyzed for cross validation purposes in Study 3, as well as basic sociodemographic questions. Therefore, the MBI:PAT consisted of the 24 items that were selected from Study 2, across six domains comprising 24 key features (see Online Resource 6). Items were presented in a random order to minimize the impact of participant completion fatigue. Participants were instructed to indicate how true each statement was, using a 6-point Likert-type scale ranging from 1 (*Not at all*) to 6 (*Outstanding*).

#### Data Analyses

Item distributions were described using means (SDs), skewness, kurtosis, and medians. The polychoric correlation matrix of selected items was calculated. The internal consistency was further determined by calculating McDonald’s (ω), with coefficients ≥ 0.70 indicating adequate internal consistency values (McDonald, 2013). The one-dimensional structure of the MBI:PAT was tested using CFA with the WLSMV estimator for ordinal variables. CFA is the recommended approach for confirming hypotheses about a preconceived factor structure (Costello & Osborne, 2005). We adopted a design-based approach. We used the group-level sampling design and adjusted standard errors of parameter estimates by means of cluster-robust standard errors via the sandwich algorithm. We used the following goodness-of-fit indices with their respective cut-off points (Schermelleh-Engel et al., 2003): CFI =  ≥ 0.95 for good and ≥ 0.90 for acceptable; TLI =  ≥ 0.95 for good and ≥ 0.90 for acceptable; RMSEA =  ≤ 0.06 for good and ≤ 0.10 for acceptable (with its 90% CI); and SRMR =  ≤ 0.06 for good and ≤ 0.10 for acceptable. Standardized factor loadings (*λ*), the uniqueness terms (*δ*), and the average variance extracted (AVE) were also estimated. Finally, we calculated the construct replicability (*H*), with *H* ≥ 0.70 indicating a reasonable definition and *H* ≥ 0.80 suggesting an adequately defined construct (Hancock & Mueller, 2001). Mplus v8.10 package was used for the analyses.

### Results

Table 2 shows the descriptive statistics (means, *SD*s, skewness, kurtosis, and medians) for the final set of 24 MBI:PAT items in the cross-validation sample, showing the item distributions. Given the one-factor solution, we also computed the descriptive statistics for the total scale score (*M* = 5.34; *SD* = 0.68), The mean inter-item polychoric correlation of the 24 items *r* = 0.7, met established benchmarks for strong internal consistency.

The analyses suggested a one-factor solution revealed with good fit (CFI = 1.00; TLI = 1.00; RMSEA = 0.04, 90% CI [0.03, 0.05]; SRMR = 0.03). Online Resource 9 shows the polychoric correlation matrix of the final set of 24 MBI:PAT items. The standardized factor loadings were higher than 0.74, and the uniqueness terms were lower than 0.45 (Table 2). The AVE, internal consistency, and construct reliability values were all high (AVE = 0.80; the internal consistency (*ω*) = 0.99; and construct replicability (*H*) = 0.99). All these results confirmed the one-factor structure and the adequate internal consistency of the 24 item MBI:PAT measure.

### Discussion

The results of Study 3 confirmed the one-factor structure of the MBI:PAT, as hypothesized, with strong psychometric properties. The CFA showed excellent goodness-of-fit indices, indicating that the 24 items measured a single latent construct (i.e., MBI delivery in terms of teaching competence (Durlak & DuPre, 2008; Tudor et al., 2022) consistently across participants. The high internal consistency, construct replicability, and average variance extracted suggests that the MBI is a reliable and valid tool for assessing MBI teaching competence.

## Study 4

Having established the MBI-PAT’s strong psychometric properties Study 4 aimed to: (i.) assess the magnitude of any teacher-level effects (i.e., potential impact of data clustering), including a comparison of factor loadings from the two-level model and the design-based approach using robust estimations; (ii.) examine the consistency of the measurement across various subgroups of participants, including those based on age, gender, and across different sub-samples (i.e., calibration, validation); and (iii.) investigate the presence of extreme scores at the lowest or highest ends of the measurement scale, to assess potential floor and ceiling effects.

### Method

#### Participants

Study 4 was conducted using the combined samples from Studies 2 and 3 (i.e., calibration and validation samples merged). The participant characteristics of Study 4 (i.e., combined sample) are shown in Table 1. Participants (*n* = 400) had a mean age of 46.50 years (*SD* = 12.10); 53.30% were female, 49.30% married, 31.80% with no dependents, 33.80% had occupations in the services sector and 30.90% in the management sector, and they presented a median of 8 (interquartile range: [8, 9]) mindfulness sessions received. The MBI most frequently received was MBCT-L (75.50%), and most MBIs were carried out synchronously online (99.30%) and were delivered by the Oxford Mindfulness Foundation (95.50%). A total of 32 certified and experienced teachers, who met good practice criteria as set out by the BAMBA (https://bamba.org.uk/), were involved in teaching the mindfulness interventions and were rated by participants.

#### Procedure

Participants used an anonymous online survey in Qualtrics (www.qualtrics.com). The survey contained the total set of MBI:PAT items and sociodemographic questions.

#### Measures

The 24 MBI:PAT items used in Study 4 to assess MBI teaching competence from the participant’s perspective were the same as those used in Study 3. Participants indicated how true each statement was using a 6-point Likert scale ranging from 1 (*Not at all*) to 6 (*Outstanding*). The internal consistency of the MBI:PAT was excellent at (ω) = 0.99.

#### Data Analyses

To assess the potential impact of teacher level data clustering, we computed a two-level maximum model (i.e., a model where the full rank of variance–covariance matrix is estimated between and within levels) (Hox et al., 2017; Wu & Kwok, 2012). For that, we included a saturated between-level component by estimating the complete rank of the between-level variance–covariance matrix and incorporated the one-factor model at the within-level. This modelling approach is recommended when the number of between-level sampling units is < 40 (Wu et al., 2017). The use of this technique allowed us to assess the extent of conflated fixed and random effect estimates for the factor loadings, which may arise due to the use of a design-based approach, even under the use of cluster-robust standard errors. The (two-level) maximum model approach circumvents the assumption of uniform structures and magnitudes of factor loadings across distinct hierarchical (between and within) levels. Furthermore, it enables the calculation of the degree of data clustering by means of the intraclass correlation coefficient (ICC).

The consistency of the measurement across subgroups of participants was established by testing the configural, metric, and scalar invariance. Configural invariance involves establishing that the underlying structure of the model (e.g., one-factor) is similar across subgroups. Metric invariance entails establishing that the relationships between items and the latent factor (i.e., factor loadings) are equivalent across subgroups. Scalar invariance involves establishing that item intercepts are also equivalent across subgroups (and therefore, it can be followed that potential observed differences between subgroups are not due to variations in how items are scored or where they start on the scale). The configurational, metric, and scalar invariance of the 24 item MBI:PAT across sub-samples (i.e., calibration, validation), and relevant sociodemographic factors that are recommended in validation studies (Ayman & Korabik, 2010) such as gender (male, female) and age (using the median to split the sample in two halves: ≤ 47.00 years, > 47.00 years), were sequentially compared and evaluated. For that, we used nested models and CFA with the WLSMV estimator for ordinal variables, along with cluster-robust (sandwich) standard errors. We considered changes in *χ*^2^ and, owing to the sensitivity to the sample size to these changes (Hair, 2009), we also ensured that both decreases in CFI and TLI, as well as increases in RMSEA and SRMR, were ≤ 0.01 and ≤ 0.02, respectively (Chen, 2007). Because the goodness-of-fit indices corrected for parsimony (e.g., RMSEA) can be improved with the addition of model constraints, these improvements were considered to be random.

The presence and absence of extreme scores was examined by investigating the potential floor and ceiling effects. For that, we calculated the percentage of respondents who assigned the highest and lowest possible scores in the MBI: PAT. It is recommended that less than 15% of the sample should receive the highest or lowest score (Terwee et al., 2007). Analyses were conducted in the Mplus v8.10 and SPSS v29 packages.

### Results

The clustering effect on the 24 MBI:PAT items was small, with ICCs ranging from < 0.00 to 0.04 meaning that there was little variance due to grouping or cluster effects. This result suggests that most of the variation in the data was not due to systematic differences between teacher level clusters, but it was due to individual factors within groups or units of observation.

The two-level maximum model approach provided similar (within-level) factor loading estimates compared to the design-based approach with the total sample (see Online Resource 10). Therefore, there was no evidence of conflated fixed and random effects because of the use of the design-based approach in our previous exploratory and confirmatory analyses, carried out in Studies 2 and 3.

The one-factor MBI:PAT model was used to explore the configural, metric, and scalar invariance of the questionnaire across sub-samples (*n*: calibration = 201, validation = 199), and sub-groups of gender (*n*: male = 80, female = 213), and age (*n*: ≤ 47.00 years = 155, > 47.00 years = 142). As can be seen in Table 3, none of the increasingly restrictive nested models of invariance (i.e., configural, metric, and scalar) exceeded cut-off recommendations (e.g., *p*-value for the Δ*χ*^2^, and ΔCFI, ΔTLI, ΔRMSEA, and ΔSRMR). At the same time, with respect to sub-samples (calibration and validation sub-samples) and sub-groups (by gender and age), there was a reasonable level of approximate fit to the data and measurement invariance, which means consistency of the measurement across the mentioned sub-samples and subgroups.

**Table 3:** Analyses of invariance according to sub-sample, gender, and age

Model	CFI	TLI	RMSEA (90% CI)	SRMR	*p*	ΔCFI	ΔTLI	ΔRMSEA	ΔSRMR
Sub-sample (calibration vs. validation)									
*Configural*	0.98	0.98	0.06 (0.06, 0.07)	0.04	_a	_a	_a	_a	_a
*Metric*	0.98	0.98	0.06 (0.06, 0.07)	0.07	0.31	0.00	−0.00	−0.00	+ 0.04
*Scalar*	0.98	0.98	0.06 (0.05, 0.07)	0.08	0.12	−0.00	−0.00	−0.00	+ 0.01
Gender (males vs. females)									
*Configural*	0.95	0.95	0.08 (0.07, 0.09)	0.04	_a	_a	_a	_a	_a
*Metric*	0.95	0.95	0.08 (0.07, 0.09)	0.07	0.61	+ 0.00	+ 0.01	−0.00	+ 0.03
*Scalar*	0.95	0.95	0.08 (0.07, 0.08)	0.08	0.44	−0.00	+ 0.00	−0.00	+ 0.00
Age (≤ 47 years vs. > 47 years)									
*Configural*	0.96	0.96	0.08 (0.07, 0.08)	0.04	_a	_a	_a	_a	_a
*Metric*	0.97	0.97	0.07 (0.07, 0.08)	0.07	0.65	+ 0.00	+ 0.00	−0.00	+ 0.03
*Scalar*	0.96	0.96	0.07 (0.06, 0.08)	0.08	0.06	−0.00	−0.00	−0.00	+ 0.00

^a^ Least restrictive model used as a reference. *p* = *p*-value for the corresponding (*X*^2^) nested comparison, *CFI* comparative fit index, *TLI* Tucker–Lewis index, *RMSEA* root-mean-square error of approximation. SRMR = standardized root mean square residual

Finally, while no participants obtained the lowest possible score (i.e., 24), 14.70% obtained the highest possible score (i.e., 144). Although both values fall within the recommended range of less than 15% according to Terwee et al (2007), this pattern may reflect potential difficulties in capturing variability in responses at the lower end of the MBI:PAT scale.

### Discussion

All these results indicate that our design-based analytical approach utilized in Studies 2 and 3, using cluster-robust standard errors, was not substantially affected by the conflation of fixed and random effect estimates for the factor loadings (Hox et al., 2017; Wu & Kwok, 2012). Our results also show that the between-cluster variation was relatively small compared to the total variation observed in the MBI:PAT items (ICCs ≤ 0.04). In addition, the one-factor MBI:PAT scale was consistent in terms of measurement across males/females, and people of different ages. Finally, the pattern of scores obtained may suggest difficulties in capturing variability in responses at the lower end of the scale (Terwee et al., 2007). However, given the small clustering effects observed, this could also be due to the limited diversity in the sample of rated MBI teachers who were all highly qualified and experienced.

## Study 5

Having further established the robustness of the MBI:PAT across score ranges and different groups, the objective of Study 5 was to assess the degree to which the 24 item MBI:PAT total score converges/diverges in relation to the following measures: (i.) expectations regarding the MBI before starting and credibility of the MBI following participation; (ii.) mental well-being before and after participating in the MBI; and (iii.) overall health before participating in the MBI, and distressing unpleasantness resulting from the MBI.

Based on previous research (Montero-Marin et al., 2021), we expected that participants' initial expectations about MBIs prior to their active involvement might contribute to subsequent improvements in their outcomes (i.e., mental well-being). In parallel, we expected that participants' ratings of the program's credibility following their engagement would be associated with improvements in health-related outcomes (Montero-Marin et al., 2021). We therefore hypothesized a positive correlation between expectations and the subsequent evaluation of MBI teaching competence using the MBI:PAT, and positive correlations between credibility and the MBI: PAT.

We also hypothesized that a higher level of MBI teaching competence, using the new MBI:PAT measure post-intervention, would be positively associated with an improvement in participants’ outcome (i.e., mental well-being) from pre- to post-intervention. This hypothesis is based on the finding that appropriate engagement with MBIs teachings and practices can enhance mental well-being (Chiesa & Serretti, 2011; Galante et al., 2021; Goldberg, 2022; Khoury et al., 2013).

Furthermore, we hypothesized that participants who perceive MBI teaching competence more positively using the new MBI:PAT measure would also report better mental health before participating in the program. This would likely be due to the effects of their baseline health status and program engagement. We also anticipated that a positive perception of MBI teaching would have an inverse relationship with reported distressing unpleasantness from the MBI. Previous research has suggested potential causal links between mindfulness practice and deviations in the subjective experience of waking consciousness (Galante et al., 2024), and specifically, it has been emphasized the importance of monitoring and addressing unpleasant thoughts, feelings, and upsetting experiences, especially when implementing MBIs in health-related contexts (Baer et al., 2021). While MBIs require both commitment and working with difficulties, we nevertheless expect that an intervention that is highly rated by participants will also be perceived as less distressing.

### Method

#### Participants

Participants’ characteristics for individuals who participated in the MBCT-TiF intervention are shown in Online Resource 11. Participants (*n* = 71) had a mean age of 51.60 (*SD* = 12.40) years, 70.40% were female, 71.80% employed, 70.40% from the UK, and 73.20% had received a previous MBCT program, while 26.80% had received a previous MBSR program. This sample size allowed us to detect small effects (Cohen’s *d* = 0.33 in a pre-post within-group analysis design, and *r* = 0.33 in a cross-sectional analysis design under the null hypothesis of *r* = 0), with a two-sided *alpha* of 0.05, and a statistical power of *beta* = 0.80. The mean (*SD*) number of years since their previous MBI was 3.85 (5.53). They received a median of 11 (interquartile range: [11, 12]) MBCT-TiF sessions. The MBCT-TiF program was delivered entirely online, in synchronous sessions, by the Oxford Mindfulness Foundation. A total of five certified and experienced MBI teachers, who met good practice criteria according to the BAMBA (https://bamba.org.uk/), were involved in teaching MBCT-TiF and were rated by participants, using an anonymous online survey in Qualtrics (www.qualtrics.com).

#### Procedure

Participants were recruited and randomized in two study cohorts, as part of a randomized controlled trial (RCT), evaluating the extent to which MBCT-TiF may improve mental well-being compared to ongoing mindfulness practice (Maloney et al., 2024). Simple randomization involved a computer-generated list (1:1) to MBCT-TIF or ongoing mindfulness practice for Cohort 1 and Cohort 2. Only the MBCT-TiF group was used in the present study. Both cohorts were recruited online through email invitation and social media posts, using the Oxford Mindfulness Foundation newsletter and social media platforms, and through an existing participant database of MBI graduates, who expressed an interest in taking part in research studies. The first study cohort was recruited and enrolled in June-2021 and the second study cohort was recruited in September-2021 and enrolled in October-2021. Further study details can be found in Maloney et al. (2024). The original trial was reviewed and approved by the Central University Research Ethics Committee at the University of Oxford (R75514/RE001; 12/05/2021). All study participants provided written informed consent prior to the start of the study. The trial was registered in ClinicalTrials.Gov (reference: NCT05154266; 13/12/2021).

#### Measures

The survey contained basic sociodemographic questions as well as the total (i.e., 73) set of MBI:PAT items and other questionnaires. The fit indices of the measurement models for the instruments used in Study 5 are presented in Online Resource 12.

The final set of 24 MBI:PAT items used in Study 5 analyses to assess MBI teaching competence from the participant’s perspective was the same as in Study 4. Participants were asked to assess each statement using a 6-point Likert scale, ranging from 1 (*Not at all*) to 6 (*Outstanding*). The internal consistency of the MBI:PAT in the Study 5 new sample of participants was excellent, with *ω* = 0.98.

Expectations and credibility were assessed at pre- and post-intervention respectively. We used five questions that measure the degree to which participants believe that the intervention is effective in improving outcomes (Montero-Marin et al., 2021). These unidimensional scales use a Likert scale that ranges between 0 (*Not at all*) and 10 (*A great deal*). To measure expectancy, this scale was implemented at pre-intervention (e.g., “How confident are you that this course will make sense to you?”). To measure credibility of the MBI, this scale was also implemented post-intervention (e.g., “How much did what was taught in the course make sense to you?”). The one-factor structure of both the expectancy and credibility scales showed adequate goodness-of-fit indices for both scales (see Online Resource 12). The internal consistency value (*ω*) obtained for expectancy at pre-intervention was excellent, at *ω* = 0.92. The internal consistency value obtained for credibility at post-intervention was excellent at ω = 0.96.

Mental well-being was assessed at pre- and post-intervention using the Warwick-Edinburgh Mental Well-being (WEMWBS) 14-item questionnaire. The WEMWBS was developed as a unidimensional tool to evaluate mental well-being in the general population and covers subjective well-being and psychological functioning (Tennant et al., 2007). Items are worded positively (e.g., “I’ve been feeling optimistic about the future”) and are answered on a scale from 1 (*None of the time*) to 5 (*All of the time*). A total score that ranges 0–40 is interpreted as probable mental health difficulties; 41–44 as possible mental health difficulties; 45–59 as average mental health; and 60–70 as high well-being. The one-factor structure of the WEMWBS had adequate goodness-of-fit indices in our study (see Online Resource 12). The internal consistency obtained was excellent at *ω* = 0.95 at pre-intervention and excellent at *ω* = 0.97 at post-intervention.

Overall health at pre-intervention was measured using a single item, taken from the WHOQOL-BREF questionnaire (World Health Organization, 1996). The single item was the following: “How satisfied are you with your health?” This item is answered on a Likert scale from 1 (*Very dissatisfied*) to 5 (*Very satisfied*). Distressing unpleasantness (Baer et al., 2021) during the MBI-TiF course was measured at post-intervention using a single item (e.g., “The mindfulness course can lead to you having unpleasant thoughts or feelings, how upsetting were these experiences?”), which was answered on a Likert scale from 0 (*Not at all*) to 3 (*Extremely*).

#### Data Analyses

Before examining the MBI:PATs convergent/discriminant validity we described the sample’s well-being and response to an MBI. We then evaluated the relationships between the MBI:PAT total score and pre-intervention measures of expectations, mental well-being, and overall health, as well as measures of credibility, mental well-being, and distressing unpleasant experiences at post-intervention. To accomplish this, we used structural equation modelling and the WLSMV estimator and computed cluster-robust standard errors (as described above). Initially, we computed the raw correlations between the MBI:PAT total score and expectations, credibility, well-being (at pre- and post-intervention), overall health, and distressing unpleasant experiences. Subsequently, we examined the relationships between the MBI:PAT total score and credibility at post-intervention, while adjusting for pre-intervention expectations (Model A). This approach aims to examine whether teaching competence, as measured by the MBI:PAT, is related to post-intervention credibility, regardless of the initial expectations of participants, providing a more precise evaluation. We also explored the relationships between the MBI:PAT total score and mental well-being at post-intervention, while controlling for pre-intervention mental well-being (Model B). This model showed whether perceived better competence of program delivery, as measured by the MBI:PAT, is related to further improvements in participants' mental well-being beyond their baseline levels. Finally, we calculated the relationships between the MBI:PAT total score and distressing unpleasant experiences at post-intervention, after accounting for pre-intervention overall health (Model C). This model aims to evaluate whether higher perceived competence of program delivery, as measured by the MBI:PAT, is associated with a reduced likelihood of encountering distressing unpleasant experiences, irrespective of the initial general health status. We calculated standardized slopes (*β*), 95% confidence intervals (*95% CIs*), and the corresponding model fit indices described above. We used a two-sided test with a 0.05 significance level. The Mplus v8.10 package was used to carry out the analyses.

### Results

Before addressing our research hypotheses, we characterized the sample in terms of well-being, measures of convergent and discriminant validity, and their response to an MBI. Participants who received the MBCT-TiF intervention showed a mean (*SD*) score of mental well-being (i.e., WEMWBS) at pre-intervention of 46.13 (7.73), which was in the average range, but closer to the lower bound of this range. After receiving the MBCT-TiF intervention, the mean (*SD*) score for mental well-being (i.e., WEMWBS) significantly improved to 52.31 (9.47), indicating a large effect (*d* = 1.02; *95% CI* [0.67, 1.37]; *p* < 0.001). Although still within the average range, the mean score surpassed the average UK population mean (*M* = 51.00, *SD* = 7.00) (Tennant et al., 2007). The mean (*SD*) score of expectations (range: 0–10) was 7.95 (1.44); the mean (*SD*) score of credibility (range: 0–10) was 8.57 (1.44); the mean (*SD*) score of overall health (range: 0–5) was 3.20 (1.02); and the mean (*SD*) score of distressing unpleasant experiences (range: 0–3) was 0.47 (0.58), with 57.50% of the group reporting “*not at all*”, 38.40% reporting “*somewhat*”, 4.10% reporting “*quite a bit*”, and no one (0.00%) reporting “*extremely*”. Results of the original trial are presented in Maloney et al. (2024).

In line with our hypotheses and as shown in Online Resource 13, we observed small or null relationships between the participants’ rating of teaching competence (MBI:PAT total score) and the pre-intervention measures of expectations (*r* = 0.12; *p* = 0.02), mental well-being (*r* = 0.04; *p* = 0.56), and overall health (*r* = 0.02; *p* = 0.62). At post-intervention, we observed large and significant relationships with credibility (*r* = 0.69; *p* < 0.001), small-to-intermediate significant relationships with mental well-being (*r* = 0.22; *p* < 0.001) and large and significant inverse relationships with distressing unpleasant experiences (*r* = −0.39; *p* < 0.001). In line with our hypotheses, and as shown in Table 3, the relationship between the MBI:PAT total score and credibility at post-intervention, while adjusting for pre-intervention expectations (Model A), was large and significant (*β* = 0.80; *p* < 0.001). The relationship between the MBI:PAT total score and mental well-being at post-intervention, after adjusting for pre-intervention mental well-being (Model B), was intermediate and significant (*β* = 0.34; *p* < 0.001). Additionally, the relationship between the MBI:PAT total score and distressing unpleasant experiences at post-intervention, after accounting for pre-intervention overall health (Model C), was inverse, large, and significant (*β* = −0.53; *p* < 0.001).

### Discussion

Consistent with our hypotheses, the results of Study 5 provided evidence for the convergent and discriminant validity of the MBI:PAT as a measure of MBI teaching competence. While pre-intervention measures, such as expectations and mental well-being, showed small or null correlations with MBI:PAT scores, post-intervention measures revealed important relationships. Specifically, high MBI:PAT scores were strongly associated with post-intervention credibility, moderately associated with improvements in mental well-being, and inversely related to distressing unpleasant experiences. In line with previous research (Galante et al., 2021; Goldberg, 2022), these findings suggest that participants' perceptions of teaching competence are more aligned with their experiences during and after the intervention, rather than their initial expectations (Montero-Marin et al., 2021). Moreover, the negative correlation with distressing unpleasant experiences supports the hypothesis that competent teaching minimizes adverse effects during MBIs (Baer et al., 2021). To address this future research could examine the relationship adherence/competence assessed by an observer rated scale such as the MBI:TAC (Table 4).

**Table 4:** Testing the convergent validity of the MBI:PAT: adjusted models

	*β*	95% CI	*p*
Model A			
Expectations (pre-)	−0.24	−0.35, −0.12	< 0.001
Credibility (post-)	0.80	0.74, 0.86	< 0.001
Model B			
Mental well-being (pre-)	−0.17	−0.38, 0.03	0.102
Mental well-being (post-)	0.34	0.17, 0.50	< 0.001
Model C			
Overall health (pre-)	0.02	−0.17, 0.17	0.973
Distressing unpleasantness (post-)	−0.53	−0.84, −0.22	< 0.001

*n* = 71 (sub-sample of participants that received MBCT-TiF). *β* = standardized slope. 95% CI = 95% CI. Fit indices of the models: Model A (CFI = 0.99, TLI = 0.99, RMSEA (90%) = 0.05 (0.03, 0.06), SRMR = 0.08); Model B (CFI = 0.97, TLI = 0.97, RMSEA (90%) = 0.04 (0.03, 0.05), SRMR = 0.10); Model C (CFI = 0.99, TLI = 0.99, RMSEA (90%) = 0.04 (0.02, 0.06), SRMR = 0.07). CFI = comparative fit index. TLI = Tucker–Lewis index. RMSEA = root-mean-square error of approximation. SRMR = standardized root mean square residual

## General Discussion

This paper outlines the development of a new measure of participants' perspective on the fidelity of MBI delivery, specifically assessing the competence of MBI teaching (MBI:PAT), and evaluates its preliminary psychometric properties. While MBIs are effective (van Agteren et al., 2021) and work through their hypothesized mechanisms (Maloney et al., 2024), this study establishes the fidelity of MBIs by providing an invaluable new measure of participants’ perspectives of how MBIs are delivered and received (Bellg et al., 2004; Kechter et al., 2019). The MBI:PAT was developed through a process of generating items that map onto established domains of MBI teaching competence and item refinement using an expert panel and feedback from former participants of MBI courses. The resultant brief 24-item measure covers the established domains and most key features of MBI competence (Crane et al., 2013), but from a participants’ perspective.

The initial development, refinement, and pilot testing phase demonstrated the items’ acceptability, face validity and reliability, and was used to reduce the measure’s length. Further field testing, using the reduced 24-item measure, demonstrated convergent and discriminant validity. As hypothesized in Study 3, the results from the field testing demonstrated that the MBI:PAT has a unidimensional structure, whereby participants’ views of teaching converged into one general competence dimension. The MBI:PAT is theoretically aligned with the MBI: TAC’s six domains, but it does not include all the key features, as some of them rely on expert knowledge (e.g., key learning and particular elements for each practice). The MBI: TAC has demonstrated to be a reliable and valid tool for assessing the fidelity of MBIs with a solid theoretical foundation (Crane et al., 2013).

In Study 4 clustering effects across the 24 MBI:PAT items were minimal (ICCs ≤ 0.04), indicating that most variance reflected individual differences rather than group-level structure. Consistent factor loadings across both two-level and design-based models support the validity of prior analyses (Studies 2 and 3), and the one-factor model demonstrated acceptable measurement invariance across calibration/validation samples, gender, and age. However, there was limited variability at the lower end of the scale, possibly due to sample homogeneity.

As hypothesized in Study 5, participants’ expectations about MBIs before starting the program were modestly associated with their post-intervention assessment of teaching competency. We also observed a large positive correlation between participants’ views of the credibility of the MBI at post-intervention and evaluations of teaching competency. However, in the corresponding multivariable model, including both expectations before the intervention and credibility afterwards, we observed that the sign of relationships reversed for expectations, with small-to-intermediate effects, while the large positive relationship for credibility was enhanced. This underscores the importance of effectively managing expectations before embarking on an MBI, as expectations may impact how the intervention is received (Montero-Marin et al., 2021).

We also hypothesized in Study 5 that participants' assessment of MBI teaching would be positively associated with outcomes in terms of mental well-being. As observed in the corresponding multivariable model, we found a moderate and positive relationship between MBI evaluation and pre-post intervention changes in mental well-being. This suggests that participants' assessment of teaching quality reflects pre-post enhancements in mental well-being, elucidating a portion of the variability in improvement is associated with the perceived competence of MBI teaching from the participants' perspective. A previous study with patients with recurrent depression in remission found no robust effects of teacher competence, as measured by the MBI:TAC tool, on outcome variables after receiving MBCT for preventing recurrent depression (Huijbers et al., 2017). Future research on the predictive validity of the MBI:TAC vs. MBI:PAT on health outcomes, using diverse samples is necessary to replicate findings.

Finally, we explored the relationship between participants’ reports of distressing unpleasant experiences during the MBI and their evaluation of teaching competence using the MBI:PAT. Again, as hypothesized, participants who reported more distressing unpleasant experiences during the MBI also rated the teaching received more negatively, albeit with very low base rates of distressing unpleasant experiences. This result underscores the importance of proper management of these types of negative experiences by the person delivering the MBI (Baer et al., 2021).

### Limitations and Future Directions

Our research had a limited sample of participants receiving an MBI and, importantly, it comprised a small number of well-trained and experienced teachers who met good practice criteria set out by the BAMBA (https://bamba.org.uk/). This is related to our finding that teacher-level clustering was minimal (ICC ≤ 0.04.This restricted our ability to explore a broad range of MBI teaching competence, especially teachers with little experience or training.

We conducted some of our analyses using the reduced set of 24 items within the context of the total pool of 73 items. Evaluating a shorter version as part of the complete item pool may introduce some bias to the results. This potential bias arises because the position of each item, along with participants' responses to all other items in the total pool, could influence their responses to the items in the reduced version, thereby enhancing item comprehension.

Further research is needed to establish test–retest reliability and sensitivity to change over the course of MBI teacher experience, as well as to conduct more extensive tests of convergent and discriminant validity using expert perspectives of MBI teaching competence (Crane & Kuyken, 2019), potential MBI mechanistic factors (Maloney et al., 2023, 2024), and other different outcomes. Future research utilizing the final set of 24 selected items is necessary to assess their psychometric properties independently of any additional items (Lantos et al., 2023). Moreover, research is needed to investigate whether and to what extent using the 24 items only decreases the high reliability values observed in the present study.

Previous research has found teaching competency in school-based mindfulness training, as rated by independent experts, to be associated with how much students practice mindfulness exercises (Montero-Marin et al., 2023). Given the centrality of mindfulness practice in MBIs, this important relationship should also be investigated using participant perspectives on MBI teaching competence (i.e., using the MBI:PAT questionnaire). Finally, it will be important to include participants from a wider range of social and cultural backgrounds, to address how questions of MBI diversity and inclusion are reflected in ratings of MBI teaching quality. Specifically, whether MBI teaching addresses inclusion, diversity, and equality (DeLuca et al., 2018); whether MBIs cause harm and altered states of consciousness (Baer et al., 2021; Galante et al., 2024), and whether MBI participants’ values and practices, e.g., around religion, are respected (Grossman, 2015). To address this broader set of contexts and range of teachers, future studies require larger samples, more nesting structures of teachers and participants from different socio-economic, cultural, racial, and gender backgrounds, and multilevel analytic approaches.

Trainees actively engaged in developing their mindfulness teaching skills may benefit from additional pedagogical input on the practical aspects of delivering an MBI, informed by feedback from participants through the MBI:PAT (Bowden et al., 2021). This could facilitate the exploration of the relationship between teacher, participant, and MBI characteristics, helping to determine what works for whom and how (Montero-Marin et al., 2024). Additionally, it may highlight which teaching aspects are most important to participants, enhancing instruction and through this enhancing MBI well-being outcomes (Maloney et al., 2024).

Finally, we did not triangulate observer (MBI:TAC) and participant (MBI:PAT) ratings. Further research is needed to examine the extent to which the MBI:TAC and MBI:PAT converge in terms of their factorial structure (e.g., unidimensional) and whether potential divergences are product of the assessor-type (i.e., experts with a deep understanding of domains and key features, or novices). This work would also form the basis for developing norms for both observer and participant ratings of teaching competence, that can be used in quality assurance, training teachers, and research.

### Conclusions and Implications

The development of the MBI:PAT builds on extensive work to establish the fidelity of MBIs through the MBI: TAC measure (Crane et al., 2013), by adding a participant version. Our work followed a systematic and pragmatic approach and provides strong preliminary support for the reliability and validity of the MBI:PAT. It offers a promising participant-centric approach to assessing MBI teaching competence, revealing preliminary evidence of acceptability, reliability, and validity, and suggesting a unidimensional structure aligning with participants' views. A copy of the measure is included in Appendix A. This provides a tool to support MBI teachers, trainers, and researchers, supplementing extant measures of fidelity, and opening avenues for further research on the causal relationships and broader implications for MBI effectiveness. If the participant and observer ratings of MBI competence offer triangulated, convergent and useful insights into MBI fidelity, this forms the basis for using the more cost-effective and efficient assessment of teaching competence.

## Supplementary Information

Below is the link to the electronic supplementary material.

Supplementary file1 (DOCX 424 KB)

Supplementary file2 (DOCX 21.1 KB)

## Data Availability

This study was not preregistered. Data are available from the corresponding author upon reasonable request.

## Declarations

### Artificial Intelligence

Artificial intelligence was not used.
