# Supplementary material for: Participants’ Perspective on the Competence of Mindfulness-Based Interventions Teaching: Development and Validation of the Mindfulness-Based Interventions-Participants’ Assessment of Teaching (MBI:PAT) Questionnaire
Source: Mindfulness (N Y). 2025 Sep 3;16(10):2825–44. doi: 10.1007/s12671-025-02668-8 (PMC12660462; doi:10.1007/s12671-025-02668-8)
Supplement: Supplementary file 1 — Supplementary file1 (DOCX 424 KB) [file 12671_2025_2668_MOESM1_ESM.docx]

**Supplemental Materials**

Participants’ perspective on the competence of mindfulness-based interventions teaching: development and validation of the Mindfulness-Based Interventions-Participants’ Assessment of Teaching (MBI:PAT) questionnaire

**Table of Contents**

page

**Online Resource 1.** Domains and key features of the MBI:TAC………………………………………….......………3

**Online Resource 2.** Content structure of the MBI:PAT (key features/items).…………………………...…...….…….4

**Online Resource 3.** Descriptive data of the total pool of items (calibration sample).……………..……...….……….6

**Online Resource 4.** Polychoric correlation: total pool of items (calibration sample).……………..………….…...….7

**Online Resource 5.** Item selection process (calibration sample).…………………………………..………....…...….8

**Online Resource 6.** Pool of finally selected items………………………………………………..…………....…….10

**Online Resource 7.** Dimensionality tests over selected items (calibration sample).…………….……….…..…...….12

**Online Resource 8.** Polychoric correlations: pool of selected items (calibration sample).…………..…….…….…..13

**Online Resource 9.** Polychoric correlations: pool of selected items (validation sample).………...……….….….….14

**Online Resource 10.** Comparison of design-based and two-level maximum model approaches (total sample)…......15

**Online Resource 11.** Participant characteristics at pre-intervention in the MBCT-TiF new sample for convergent and discriminant validity evaluation………………………………………………………..……………….….16

**Online Resource 12.** Fit indices and internal consistency for the measurement models using confirmatory factor analysis in the MBCT-TiF sub-sample………………………………………………………………….………..17

**Online Resource 13.** Convergent and discriminant validity of the MBI:PAT: raw relationships (MBCT-TiF sub-sample) ………………………………………………………………………………………………....….…….18

**Online Resource 1. Domains and key features of the MBI:TAC**

Domain 1: Coverage, pacing, and organization

*KF1 – Adherence to the form of the program.*

*KF2 – Responsiveness and flexibility.*

*KF3 –- Appropriate timing of themes and content.*

*KF4 – Level of organization of teacher, room, and materials.*

*KF5 –- The degree to which the session flows.*

Domain 2: Relational skills

*KF6 – Authenticity and potency – relating in a way that seems genuine, honest, and confident.*

*KF7 – Connection and acceptance – actively attending to, attuning to, and connecting with participants and their present moment experience, and conveying back an accurate and empathic understanding of this.*

*KF8 – Compassion and warmth – conveying a deep awareness, sensitivity, appreciation, and openness to participants’ experience.*

*KF9 – Curiosity and respect – conveying genuine interest in each participant and his/her experience, while respecting each participants’ vulnerabilities, boundaries and need for privacy.*

*KF10 – Mutuality – engaging with the participants in a mutual collaborative working relationship.*

Domain 3: Embodiment of mindfulness

*KF11 – Expression of present moment focus through behavior, verbal, and non-verbal communication.*

*KF12 – Responsiveness to the uniqueness of the present moment – the teacher works with the emergent moment.*

*KF13 – Simultaneously conveying steadiness, calm, ease, non-reactivity, alertness, and vitality.*

*KF14 – Allowing – the teacher’s behavior is non-judging, patient, accepting and non-striving.*

*KF15 – Natural presence of the teacher – the teacher’s behavior is authentic to his/her own intrinsic mode of operating.*

Domain 4: Guiding mindfulness practices

*KF 16 – Language: clear, precise, accurate, accessible, audible, and spacious*

*KF17 – Key learning for each practice available*

*KF18 – Elements to consider when guiding, including practical issues, safety, and trauma considerations*

Domain 5: Conveying course themes through interactive inquiry and didactic teaching.

*KF19* *– Experiential focus – supporting participants to notice and describe the different elements of direct experience and their interaction with each other; teaching themes are consistently linked to this direct experience.*

*KF20* *– Exploring the different layers within the inquiry process (e.g., direct experience, reflection on direct experience, and linking both to wider learning) with a predominant focus on process rather than content.*

*KF21 – Conveying learning themes through skillful teaching.*

*KF22* *– Fluency – the teacher conveys ease, familiarity with and confident knowledge of the material.*

*KF23 – Enabling learning – the teaching is effective in enabling learning to happen*

Domain 6: Holding the group learning environment.

*KF24 – Learning container – creating and sustaining a rich learning environment made safe through careful management of issues such as ground rules, boundaries, and confidentiality, but which is simultaneously a place in which participants can explore and take risks.*

*KF25 – Group development – clear management of group development processes over the eight weeks, particularly the management of beginnings, endings, and challenges from within the group.*

*KF26 – Personal to universal learning – the teacher consistently opens the learning process towards connection with the universality and common humanity of the processes under exploration.*

*KF27 – Leadership style that offers sustained ‘holding,’ and demonstrating authority and potency without imposing the teacher’s views on participants.*

**Online Resource 2. Content structure of the MBI:PAT (key features/items)**

*Domain 1: Coverage, pacing, and organization*

*KF1 – Adherence to the form of the program*

1. Guided mindfulness practices were part of the teaching.

2. The teacher encouraged practicing at home.

3. There was time each class to discuss experiences of the home practice.

4. The classes flowed well together, with each lesson topic feeling as if it followed on from the last.

5. It was clear what the topic of each class was.

*KF2 – Responsiveness and flexibility*

6. The teacher changed planned activities in response to how the lesson progressed (for example, deciding not to do a planned activity because it was no longer needed, or changing an activity to better help the class learn).

7. The teacher responded to the learning needs of the class (for example, spending more time on something to help people understand).

8. The teacher was flexible about the plan for the lesson (for example, discontinuing an activity that was not right for the learning needs of the class).

*KF3 – Appropriate timing of themes and content*

9. The teaching was pitched at the right level for me.

10. The teacher checked if participants understood what had been taught before moving on.

*KF4 – Level of organization of teacher, room, and materials*

11. Everything was ready and set up in time for each class to begin. For example, enough chairs had been put out, or if this was an online course, the teacher had the technology ready to start on time.

12. The teacher was organized.

13. Handouts and materials were provided or easy to access.

*KF5 – The degree to which the session flows*

14. The pace of the classes was right for me (for example, activities were not too rushed or too slow).

*Domain 2: Relational skills*

*KF6 – Authenticity and potency – relating in a way that seems genuine, honest, and confident.*

15. The teacher appeared to be themselves (genuine/authentic) during the classes.

*KF7 – Connection and acceptance – actively attending to, attuning to, and connecting with participants and their present moment experience, and conveying back an accurate and empathic understanding of this.*

16. I felt my teacher understood me.

17. I felt listened to by my teacher.

18. The teacher accepted me as I am.

*KF8 – Compassion and warmth – conveying a deep awareness, sensitivity, appreciation, and openness to participants’ experiences.*

19. I felt able to talk to the teacher about thoughts and feelings that came up during classes.

20. The teacher responded warmly and kindly when participants shared thoughts or feelings.

21. I felt the teacher cared about me.

22. The teacher helped me to feel safe in class.

23. I felt able to speak openly to the teacher.

*KF9 – Curiosity and respect – conveying genuine interest in each participant and his/her experience, while respecting each participants’ vulnerabilities, boundaries and need for privacy.*

24. The teacher treated participants with respect.

25. The teacher encouraged exploration of difficult experiences during practices.

26. The teacher seemed genuinely interested in participants’ experiences during practices.

27. The teacher encouraged participants to share only as much as they were comfortable with.

*KF10 – Mutuality – engaging with the participants in a mutual collaborative working relationship.*

28. I felt involved in the classes.

29. The classes were engaging.

30. I got the impression the teacher practiced mindfulness outside of the classes, in their everyday life.

*Domain 3: Embodying mindfulness*

*KF11 – Expression of present moment focus through behavior, verbal, and non-verbal communication.*

31. The teacher seemed to be in the present moment (in the here and now) during classes.

32. The teacher seemed to be alert during classes.

33. The teacher behaved calmly.

34. The teacher appeared to be mindful whilst teaching the classes.

*KF12 – Responsiveness to the uniqueness of the present moment – the teacher works with the emergent moment.*

35. The teacher responded to participants’ needs in the moment.

36. The teacher responded to the needs of the group in the moment.

*KF13 – Simultaneously conveying steadiness, calm, ease, non-reactivity, alertness, and vitality.*

37. The teacher responded calmly to challenging situations.

38. In challenging situations, the teacher was present (in the here and now).

*KF14 – Allowing – the teacher’s behavior is non-judging, patient, accepting, and non-striving.*

39. The teacher provided enough time to think after asking a question.

40. I felt the teacher was accepting and non-judgmental.

41. The teacher seemed at ease with themselves (for example, not being critical of themselves or trying too hard to get things “right”).

*KF15 – Natural presence of the teacher – the teacher’s behavior is authentic to his/her own intrinsic mode of operating.*

42. I got the impression that the teacher followed what they taught in class in their everyday life.

*Domain 4: Guiding mindfulness practices*

*KF16 – Language clear, precise, accessible*

43. When the teacher guided mindfulness practices, the instructions were clear.

44. When guiding mindfulness practices, the teacher’s language helped me to be aware of my senses (for example, what I could hear, see, touch, taste, or smell)

45. When guiding mindfulness practices, the teacher’s instructions about what to pay attention to were clear.

46. When guiding mindfulness practices, the teacher’s instructions helped me to understand what to do when my mind wandered.

47. The teacher reminded me in their instructions not to be critical of myself during mindfulness practices.

48. During mindfulness practices, the teacher combined guidance (instructions) with moments of silence in a helpful way (for example, not talking for a suitable amount of time between giving instructions).

49. During mindfulness practices, the teacher reminded us not to be judgmental when the mind wandered.

50. The teacher led mindfulness practices in a way that helped me to be mindful during these practices.

*Domain 5: Conveying course themes through interactive inquiry and didactic teaching*

*KF17 – Experiential focus – supporting participants to notice and describe the different elements of direct experience and their interaction with each other; teaching themes are consistently linked to this direct experience.*

51. The teacher reminded us to pay attention to emotions.

52. The teacher reminded us to pay attention to body sensations.

53. The teacher helped me learn the difference between thoughts, emotions, and body sensations.

54. The teacher helped me understand how awareness of thoughts, feelings and body sensations can be helpful.

*KF18 – Exploring the different layers within the inquiry process (e.g., direct experience, reflection on direct experience, and linking both to wider learning) with a predominant focus on process rather than content.*

55. The teacher helped me learn from my experiences during mindfulness practices.

56. The teacher helped me learn how mindfulness can be used in everyday life.

*KF19 – Conveying learning themes through skillful teaching.*

57. The teacher used a variety of different teaching methods (for example, flip charts, stories, poems, etc)

58. The teacher’s questions, comments, and reflections during group discussions helped my learning.

59. The teacher was effective at supporting my learning.

*KF20 – Fluency – the teacher conveys ease, familiarity with and confident knowledge of the material.*

60. The teacher was confident in their mindfulness knowledge.

61. The teacher seemed confident when teaching.

62. I got the impression the teacher believed in what they were teaching me.

*Domain 6: Holding the group learning environment*

*KF21 – Learning container – creating and sustaining a rich learning environment made safe through careful management of issues such as ground rules, boundaries, and confidentiality, but which is simultaneously a place in which participants can explore and take risks.*

63. The teacher helped me to feel safe in the group.

64. The teacher helped me to feel included in the group.

65. The teacher really respected confidentiality (keeping things private) within the group.

*KF22 – Group development – clear management of group development processes over the eight weeks, particularly the management of beginnings, endings, and challenges from within the group*

66. The teacher ensured the group kept to a set of “ground rules” throughout the classes.

67. The teacher gave everyone in the group a chance to talk.

68. The teacher dealt skillfully with challenging group members (for example, people who argued with the teacher or those who talked too much).

*KF23 – Personal to universal learning – the teacher consistently opens the learning process towards connection with the universality and common humanity of the processes under exploration.*

69. The teacher supported the group well during difficult moments.

70. The teacher helped me understand that everyone experiences challenging thoughts and emotions.

71. The teacher noticed if the group was affected by something that one person said.

*KF24 – Leadership style that offers sustained ‘holding’ and demonstrating authority and potency without imposing the teacher’s views on participants.*

72. The teacher's style of leading seemed right for the group.

73. The group seemed to respect the teacher.

**Online Resource 3. Descriptive data of the total pool of items (calibration sample)**

| **Item** | **M** | **SD** |  | **Skew** | **Kurt** | **Median** |
| --- | --- | --- | --- | --- | --- | --- |
| **1** | 5.76 | 0.61 |  | -2.84 | 8.13 | 6 |
| **2** | 5.72 | 0.65 |  | -2.49 | 5.90 | 6 |
| **3** | 5.74 | 0.68 |  | -2.69 | 6.55 | 6 |
| **4** | 5.12 | 0.79 |  | -0.89 | 1.10 | 5 |
| **5** | 5.17 | 0.84 |  | -1.04 | 1.26 | 5 |
| **6** | 4.17 | 1.48 |  | -0.76 | -0.36 | 5 |
| **7** | 4.94 | 1.13 |  | -1.35 | 1.93 | 5 |
| **8** | 4.52 | 1.34 |  | -1.01 | 0.52 | 5 |
| **9** | 5.13 | 0.94 |  | -1.03 | 0.90 | 5 |
| **10** | 5.12 | 1.00 |  | -1.34 | 2.30 | 5 |
| **11** | 5.47 | 0.76 |  | -1.44 | 2.09 | 6 |
| **12** | 5.48 | 0.70 |  | -1.42 | 2.68 | 6 |
| **13** | 5.41 | 0.82 |  | -1.43 | 1.82 | 6 |
| **14** | 5.16 | 0.95 |  | -1.22 | 1.81 | 5 |
| **15** | 5.59 | 0.78 |  | -2.49 | 8.02 | 6 |
| **16** | 5.11 | 1.06 |  | -1.45 | 2.49 | 5 |
| **17** | 5.33 | 0.89 |  | -1.86 | 5.20 | 6 |
| **18** | 5.47 | 0.81 |  | -1.80 | 4.51 | 6 |
| **19** | 5.27 | 0.99 |  | -1.60 | 2.95 | 6 |
| **20** | 5.61 | 0.66 |  | -1.66 | 2.30 | 6 |
| **21** | 5.27 | 0.88 |  | -1.78 | 5.19 | 5 |
| **22** | 5.46 | 0.77 |  | -1.85 | 5.41 | 6 |
| **23** | 5.30 | 0.90 |  | -1.48 | 2.78 | 6 |
| **24** | 5.69 | 0.60 |  | -1.90 | 3.08 | 6 |
| **25** | 5.18 | 0.94 |  | -1.16 | 1.03 | 5 |
| **26** | 5.44 | 0.87 |  | -2.06 | 5.60 | 6 |
| **27** | 5.45 | 0.78 |  | -2.01 | 6.45 | 6 |
| **28** | 5.26 | 0.89 |  | -1.52 | 3.28 | 5 |
| **29** | 5.23 | 0.91 |  | -1.40 | 2.48 | 5 |
| **30** | 5.51 | 0.77 |  | -1.89 | 4.32 | 6 |
| **31** | 5.50 | 0.73 |  | -1.61 | 3.04 | 6 |
| **32** | 5.53 | 0.68 |  | -1.38 | 1.61 | 6 |
| **33** | 5.60 | 0.63 |  | -1.55 | 2.14 | 6 |
| **34** | 5.57 | 0.66 |  | -1.53 | 2.18 | 6 |
| **35** | 5.42 | 0.73 |  | -1.04 | 0.49 | 6 |
| **36** | 5.39 | 0.74 |  | -1.05 | 0.65 | 6 |
| **37** | 5.38 | 0.77 |  | -1.34 | 2.06 | 6 |
| **38** | 5.39 | 0.77 |  | -1.30 | 1.88 | 6 |
| **39** | 5.33 | 0.81 |  | -1.12 | 1.06 | 6 |
| **40** | 5.51 | 0.80 |  | -2.27 | 7.13 | 6 |
| **41** | 5.39 | 0.92 |  | -2.20 | 6.25 | 6 |
| **42** | 5.44 | 0.75 |  | -1.78 | 5.83 | 6 |
| **43** | 5.49 | 0.63 |  | -0.96 | 0.44 | 6 |
| **44** | 5.43 | 0.77 |  | -1.44 | 2.30 | 6 |
| **45** | 5.41 | 0.72 |  | -0.95 | 0.13 | 6 |
| **46** | 5.47 | 0.70 |  | -1.22 | 1.11 | 6 |
| **47** | 5.50 | 0.70 |  | -1.30 | 1.28 | 6 |
| **48** | 5.27 | 0.84 |  | -1.11 | 1.20 | 5 |
| **49** | 5.50 | 0.71 |  | -1.48 | 2.69 | 6 |
| **50** | 5.42 | 0.73 |  | -1.63 | 5.73 | 6 |
| **51** | 5.23 | 0.82 |  | -1.23 | 2.84 | 5 |
| **52** | 5.45 | 0.66 |  | -0.89 | 0.14 | 6 |
| **53** | 5.28 | 0.83 |  | -1.14 | 1.22 | 5 |
| **54** | 5.41 | 0.80 |  | -1.41 | 1.95 | 6 |
| **55** | 5.28 | 0.90 |  | -1.63 | 3.74 | 5 |
| **56** | 5.40 | 0.78 |  | -1.42 | 2.61 | 6 |
| **57** | 5.18 | 0.95 |  | -1.19 | 1.18 | 5 |
| **58** | 5.24 | 0.88 |  | -1.26 | 1.55 | 5 |
| **59** | 5.21 | 0.91 |  | -1.52 | 3.57 | 5 |
| **60** | 5.51 | 0.71 |  | -1.54 | 2.35 | 6 |
| **61** | 5.47 | 0.74 |  | -1.53 | 2.83 | 6 |
| **62** | 5.61 | 0.70 |  | -2.56 | 10.15 | 6 |
| **63** | 5.43 | 0.81 |  | -1.72 | 4.28 | 6 |
| **64** | 5.37 | 0.84 |  | -1.68 | 4.06 | 6 |
| **65** | 5.55 | 0.66 |  | -1.59 | 3.63 | 6 |
| **66** | 5.31 | 0.78 |  | -1.12 | 1.41 | 5 |
| **67** | 5.42 | 0.74 |  | -1.32 | 2.17 | 6 |
| **68** | 5.28 | 0.86 |  | -1.64 | 4.12 | 5 |
| **69** | 5.26 | 0.86 |  | -1.30 | 1.79 | 5 |
| **70** | 5.35 | 0.83 |  | -1.79 | 5.06 | 6 |
| **71** | 5.05 | 1.04 |  | -1.29 | 1.92 | 5 |
| **72** | 5.32 | 0.87 |  | -1.85 | 4.91 | 5 |
| **73** | 5.56 | 0.63 |  | -1.10 | 0.13 | 6 |

*Note.* M = mean. SD = standard deviation. Skew = skewness. Kurt = kurtosis.

**Online Resource 4. Polychoric correlation: total pool of items (calibration sample)**

Mean inter-item correlations = 0.69

**Online Resource 5. Item selection process (calibration sample)**

*Domain 1: Coverage, pacing, and organization.*

| Items | Corrected Item-Dom1 | Item-Dom2 | Item-Dom3 | Item-Dom4 | Item-Dom5 | Item-Dom6 |
| --- | --- | --- | --- | --- | --- | --- |
| 1 | 0.330 | 0.27 | 0.28 | 0.39 | 0.28 | 0.25 |
| 2 | 0.432 | 0.40 | 0.40 | 0.41 | 0.41 | 0.41 |
| 3 | 0.437 | 0.37 | 0.33 | 0.36 | 0.35 | 0.33 |
| **4** | **0.673** | 0.63 | 0.62 | 0.62 | 0.66 | 0.63 |
| 5 | 0.649 | 0.54 | 0.53 | 0.59 | 0.58 | 0.58 |
| 6 | 0.549 | 0.42 | 0.36 | 0.37 | 0.43 | 0.44 |
| **7** | **0.738** | 0.69 | 0.60 | 0.62 | 0.65 | 0.67 |
| 8 | 0.681 | 0.57 | 0.51 | 0.52 | 0.57 | 0.56 |
| **9** | **0.715** | 0.64 | 0.59 | 0.62 | 0.66 | 0.60 |
| 10 | 0.657 | 0.65 | 0.64 | 0.56 | 0.61 | 0.69 |
| 11 | 0.639 | 0.67 | 0.69 | 0.62 | 0.64 | 0.68 |
| **12** | **0.667** | 0.72 | 0.77 | 0.71 | 0.75 | 0.76 |
| 13 | 0.487 | 0.49 | 0.52 | 0.53 | 0.50 | 0.49 |
| **14** | **0.641** | 0.60 | 0.53 | 0.59 | 0.58 | 0.55 |

Dom: Domain. Bold: items selected according to the item-rest correlation values.

Dashed lines separate items pertaining to different key features of the same domain.

*Domain 2: Relational skills.*

| Items | Corrected Item-Dom2 | Item-Dom1 | Item-Dom3 | Item-Dom4 | Item-Dom5 | Item-Dom6 |
| --- | --- | --- | --- | --- | --- | --- |
| **15** | **0.823** | 0.70 | 0.77 | 0.68 | 0.73 | 0.72 |
| 16 | 0.831 | 0.70 | 0.73 | 0.63 | 0.69 | 0.71 |
| **17** | **0.864** | 0.70 | 0.81 | 0.69 | 0.76 | 0.80 |
| 18 | 0.851 | 0.68 | 0.80 | 0.66 | 0.71 | 0.76 |
| 19 | 0.841 | 0.67 | 0.73 | 0.63 | 0.71 | 0.75 |
| 20 | 0.794 | 0.65 | 0.78 | 0.69 | 0.70 | 0.72 |
| 21 | 0.807 | 0.67 | 0.73 | 0.65 | 0.71 | 0.72 |
| 22 | 0.847 | 0.70 | 0.83 | 0.69 | 0.73 | 0.79 |
| **23** | **0.881** | 0.69 | 0.79 | 0.72 | 0.77 | 0.80 |
| 24 | 0.794 | 0.67 | 0.78 | 0.66 | 0.68 | 0.73 |
| 25 | 0.710 | 0.64 | 0.70 | 0.66 | 0.69 | 0.74 |
| **26** | **0.816** | 0.68 | 0.77 | 0.68 | 0.79 | 0.77 |
| 27 | 0.669 | 0.64 | 0.68 | 0.66 | 0.65 | 0.67 |
| **28** | **0.772** | 0.65 | 0.69 | 0.64 | 0.70 | 0.71 |
| 29 | 0.669 | 0.72 | 0.60 | 0.63 | 0.67 | 0.61 |
| 30 | 0.658 | 0.59 | 0.70 | 0.56 | 0.64 | 0.65 |

Dom: Domain. Bold: items selected according to the item-rest correlation values.

Dashed lines separate items pertaining to different key features of the same domain.

*Domain 3: Embodiment of mindfulness.*

| Items | Corrected Item-Dom3 | Item-Dom1 | Item-Dom2 | Item-Dom4 | Item-Dom5 | Item-Dom6 |
| --- | --- | --- | --- | --- | --- | --- |
| 31 | 0.857 | 0.67 | 0.77 | 0.74 | 0.76 | 0.79 |
| 32 | 0.802 | 0.66 | 0.74 | 0.72 | 0.72 | 0.73 |
| 33 | 0.807 | 0.63 | 0.73 | 0.69 | 0.71 | 0.73 |
| **34** | **0**.**875** | 0.66 | 0.78 | 0.73 | 0.77 | 0.79 |
| 35 | 0.803 | 0.73 | 0.82 | 0.74 | 0.79 | 0.80 |
| **36** | **0.838** | 0.75 | 0.85 | 0.76 | 0.82 | 0.84 |
| **37** | 0**.891** | 0.72 | 0.81 | 0.75 | 0.84 | 0.83 |
| 38 | 0.880 | 0.72 | 0.82 | 0.76 | 0.82 | 0.82 |
| 39 | 0.791 | 0.69 | 0.77 | 0.74 | 0.76 | 0.78 |
| 40 | **0.845** | 0.66 | 0.82 | 0.70 | 0.77 | 0.79 |
| 41 | 0.759 | 0.61 | 0.74 | 0.67 | 0.71 | 0.72 |
| **42** | **0.791** | 0.61 | 0.70 | 0.69 | 0.77 | 0.72 |

Dom: Domain. Bold: items selected according to the item-rest correlation values.

Dashed lines separate items pertaining to different key features of the same domain.

*Domain 4: Guiding mindfulness practices.*

| Items | Corrected Item-Dom4 | Item-Dom2 | Item-Dom3 | Item-Dom4 | Item-Dom5 | Item-Dom6 |
| --- | --- | --- | --- | --- | --- | --- |
| 43 | 0.776 | 0.64 | 0.67 | 0.75 | 0.74 | 0.73 |
| 44 | 0.802 | 0.66 | 0.69 | 0.67 | 0.72 | 0.68 |
| 45 | 0.829 | 0.67 | 0.67 | 0.74 | 0.77 | 0.73 |
| **46** | **0.863** | 0.73 | 0.74 | 0.79 | 0.81 | 0.77 |
| 47 | 0.814 | 0.63 | 0.67 | 0.71 | 0.75 | 0.70 |
| 48 | 0.761 | 0.68 | 0.67 | 0.70 | 0.73 | 0.69 |
| 49 | 0.781 | 0.66 | 0.65 | 0.68 | 0.72 | 0.69 |
| 50 | 0.760 | 0.72 | 0.72 | 0.70 | 0.77 | 0.71 |

Dom: Domain. Bold: items selected according to the item-rest correlation values.

*Domain 5: Conveying course themes through interactive inquiry and didactic teaching.*

| Items | Corrected Item-Dom5 | Item-Dom1 | Item-Dom2 | Item-Dom3 | Item-Dom4 | Item-Dom6 |
| --- | --- | --- | --- | --- | --- | --- |
| 51 | 0.789 | 0.65 | 0.69 | 0.71 | 0.75 | 0.74 |
| 52 | 0.815 | 0.70 | 0.69 | 0.74 | 0.84 | 0.77 |
| 53 | 0.772 | 0.62 | 0.70 | 0.72 | 0.74 | 0.73 |
| **54** | **0.824** | 0.68 | 0.71 | 0.75 | 0.74 | 0.75 |
| **55** | 0**.830** | 0.69 | 0.75 | 0.73 | 0.72 | 0.79 |
| 56 | 0.821 | 0.72 | 0.78 | 0.77 | 0.75 | 0.78 |
| 57 | 0.708 | 0.67 | 0.63 | 0.65 | 0.66 | 0.75 |
| 58 | 0.871 | 0.77 | 0.82 | 0.80 | 0.77 | 0.84 |
| **59** | **0.872** | 0.77 | 0.83 | 0.82 | 0.76 | 0.85 |
| 60 | 0.769 | 0.64 | 0.64 | 0.75 | 0.73 | 0.75 |
| **61** | **0.802** | 0.68 | 0.71 | 0.81 | 0.74 | 0.78 |
| 62 | 0.775 | 0.70 | 0.75 | 0.81 | 0.73 | 0.80 |

Dom: Domain. Bold: items selected according to the item-rest correlation values.

Dashed lines separate items pertaining to different key features of the same domain.

*Domain 6: Holding the group’s learning environment.*

| Items | Corrected Item-Dom6 | Item-Dom1 | Item-Dom2 | Item-Dom3 | Item-Dom4 | Item-Dom5 |
| --- | --- | --- | --- | --- | --- | --- |
| 63 | 0.851 | 0.71 | 0.83 | 0.81 | 0.69 | 0.77 |
| **64** | **0.868** | 0.70 | 0.83 | 0.80 | 0.69 | 0.79 |
| 65 | 0.676 | 0.64 | 0.58 | 0.63 | 0.62 | 0.63 |
| **66*** | **0.756** | 0.64 | 0.65 | 0.71 | 0.69 | 0.75 |
| 67 | 0.687 | 0.63 | 0.65 | 0.67 | 0.61 | 0.64 |
| 68 | 0**.**783 | 0.65 | 0.72 | 0.75 | 0.67 | 0.78 |
| **69** | **0.842** | 0.73 | 0.80 | 0.81 | 0.77 | 0.88 |
| 70 | 0.796 | 0.67 | 0.72 | 0.77 | 0.71 | 0.84 |
| 71 | 0.823 | 0.71 | 0.79 | 0.75 | 0.70 | 0.80 |
| **72** | **0.804** | 0.76 | 0.81 | 0.79 | 0.76 | 0.81 |
| 73 | 0.735 | 0.61 | 0.68 | 0.71 | 0.71 | 0.70 |

Dom: Domain. Bold: items selected according to the item-rest correlation values. Dashed lines separate items pertaining to different key features of the same domain. *Although the item number 68 (“The teacher dealt skillfully with challenging group members (for example, people who argued with the teacher or those who talked too much”) presented the highest corrected item-rest (item-domain6) value in its corresponding key feature (i.e., Group development – clear management of group development processes over the eight weeks, particularly the management of beginnings, endings, and challenges from within the group), it was established by consensus that the item number 66 (“The teacher ensured the group kept to a set of “ground rules” throughout the classes”) would theoretically better represent this key feature. Therefore, the item number 66 was finally selected.

**Online Resource 6. Pool of finally selected items**

**(Domain 1: Coverage, pacing, and organization)**

**Key Features/Items:**

*KF1 – Adherence to the form of the program*

**Item 4**. The classes flowed well together, with each lesson topic feeling as if it followed on from the last.

*KF2 – Responsiveness and flexibility*

**Item 7**. The teacher responded to the learning needs of the class (for example, spending more time on something to help people understand).

*KF3 – Appropriate timing of themes and content*

**Item 9**. The teaching was pitched at the right level for me.

*KF4 – Level of organization of teacher, room, and materials*

**Item 12**. The teacher was organized.

*KF5 – The degree to which the session flows*

**Item 14**. The pace of the classes was right for me (for example, activities were not too rushed or too slow).

**(Domain 2: Relational skills)**

**Key Features/Items:**

*KF6 – Authenticity and potency – relating in a way that seems genuine, honest, and confident.*

**Item 15**. The teacher appeared to be themselves (genuine/authentic) during the classes.

*KF7 – Connection and acceptance – actively attending to, attuning to, and connecting with participants and their present moment experience, and conveying back an accurate and empathic understanding of this.*

**Item 17**. I felt listened to by my teacher.

*KF8 – Compassion and warmth – conveying a deep awareness, sensitivity, appreciation, and openness to participants’ experience.*

**Item 23**. I felt able to speak openly to the teacher.

*KF9 – Curiosity and respect – conveying genuine interest in each participant and his/her experience, while respecting each participants’ vulnerabilities, boundaries and need for privacy.*

**Item 26**. The teacher seemed genuinely interested in participants’ experiences during practices.

*KF10 – Mutuality – engaging with the participants in a mutual collaborative working relationship.*

**Item 28**. I felt involved in the classes.

**(Domain 3: Embodiment of mindfulness)**

**Key Features/Items:**

*KF11 – Expression of present moment focus through behavior, verbal, and non-verbal communication.*

**Item 34**. The teacher appeared to be mindful whilst teaching the classes.

*KF12 – Responsiveness to the uniqueness of the present moment – the teacher works with the emergent moment.*

**Item 36**. The teacher responded to the needs of the group in the moment.

*KF13 – Simultaneously conveying steadiness, calm, ease, non-reactivity, alertness, and vitality.*

**Item 37**. The teacher responded calmly to challenging situations.

*KF14 – Allowing – the teacher’s behavior is non-judging, patient, accepting and non-striving.*

**Item 40**. I felt the teacher was accepting and non-judgmental.

*KF15 – Natural presence of the teacher – the teacher’s behavior is authentic to his/her own intrinsic mode of operating.*

**Item 42**. I got the impression that the teacher followed what they taught in class in their everyday life.

**(Domain 4: Guiding mindfulness practices)**

**Key Features/Items:**

*KF16 – Language clear, precise, accurate, accessible*

**Item 46.** When guiding mindfulness practices, the teacher’s instructions helped me to understand what to do when my mind wandered.

**(Domain 5: Conveying course themes through interactive inquiry and didactic teaching)**

**Key Features/Items:**

*KF17 – Experiential focus – supporting participants to notice and describe the different elements of direct experience and their interaction with each other; teaching themes are consistently linked to this direct experience.*

**Item 54.** The teacher helped me understand how awareness of thoughts, feelings, and body sensations can be helpful.

*KF18 – Exploring the different layers within the inquiry process (e.g., direct experience, reflection on direct experience, and linking both to wider learning) with a predominant focus on process rather than content.*

**Item 55.** The teacher helped me learn from my experiences during mindfulness practices.

*KF19 – Conveying learning themes through skillful teaching.*

**Item 59.** The teacher was effective at supporting my learning.

*KF20 – Fluency – the teacher conveys ease, familiarity with, and confident knowledge of the material.*

**Item 61.** The teacher seemed confident when teaching.

**(Domain 6: Holding the group’s learning environment)**

**Key Features/Items:**

*KF21 – Learning container – creating and sustaining a rich learning environment made safe through careful management of issues such as ground rules, boundaries, and confidentiality, but which is simultaneously a place in which participants can explore and take risks.*

**Item 64.** The teacher helped me to feel included in the group.

*KF22 – Group development – clear management of group development processes over the eight weeks, particularly the management of beginnings, endings, and challenges from within the group.*

**Item 66.** The teacher ensured the group kept to a set of “ground rules” throughout the classes.

*KF23 – Personal to universal learning – the teacher consistently opens the learning process towards connection with the universality and common humanity of the processes under exploration.*

**Item 69.** The teacher supported the group well during difficult moments.

*KF24 – Leadership style that offers sustained ‘holding’ and demonstrating authority and potency without imposing the teacher’s views on participants.*

**Item 72.** The teacher's style of leading seemed right for the group.

**Online Resource 7. Dimensionality tests over selected items (calibration sample)**

*Schwartz’s Bayesian information criterion (BIC)*

Number of factors BIC

0 24163.363

1 397.779*

2 461.448

3 558.510

* Advised number of common factors

*Parallel Analysis (PA)*

Variable Real-data Mean of random 95 percentile of random

% of variance % of variance % of variance

1 69.2024* 8.7735 9.6651

2 4.9918 8.0872 8.8006

3 3.5352 7.5758 8.1360

4 3.1945 7.1128 7.6245

5 2.3197 6.6984 7.1350

6 1.9713 6.3077 6.6700

7 1.8667 5.9445 6.3023

8 1.8488 5.5994 5.9203

9 1.7616 5.2586 5.5924

…

* Advised number of dimensions

**Online Resource 8. Polychoric correlations: pool of selected items (calibration sample)**

 Mean inter-item correlations = 0.74

**Online Resource 9. Polychoric correlations: pool of selected items (validation sample)**

|  | **4** | **7** | **9** | **12** | **14** | **15** | **17** | **23** | **26** | **28** | **34** | **36** | **37** | **40** | **42** | **46** | **54** | **55** | **59** | **61** | **64** | **66** | **69** | **72** |
| --- | --- | --- | --- | --- | --- | --- | --- | --- | --- | --- | --- | --- | --- | --- | --- | --- | --- | --- | --- | --- | --- | --- | --- | --- |
| **ITEM4** | 1 |  | | | | | | | | | | | | | | | | | | | | | | |
| **ITEM7** | 0.67 | 1 |  | | | | | | | | | | | | | | | | | | | | | |
| **ITEM9** | 0.69 | 0.67 | 1 |  | | | | | | | | | | | | | | | | | | | | |
| **ITEM12** | 0.60 | 0.62 | 0.63 | 1 |  | | | | | | | | | | | | | | | | | | | |
| **ITEM14** | 0.69 | 0.64 | 0.74 | 0.72 | 1 |  | | | | | | | | | | | | | | | | | | |
| **ITEM15** | 0.67 | 0.69 | 0.78 | 0.76 | 0.75 | 1 |  | | | | | | | | | | | | | | | | | |
| **ITEM17** | 0.71 | 0.71 | 0.79 | 0.69 | 0.76 | 0.84 | 1 |  | | | | | | | | | | | | | | | | |
| **ITEM23** | 0.64 | 0.71 | 0.73 | 0.66 | 0.68 | 0.82 | 0.88 | 1 |  | | | | | | | | | | | | | | | |
| **ITEM26** | 0.70 | 0.66 | 0.74 | 0.71 | 0.69 | 0.83 | 0.86 | 0.82 | 1 |  | | | | | | | | | | | | | | |
| **ITEM28** | 0.72 | 0.73 | 0.80 | 0.68 | 0.73 | 0.76 | 0.87 | 0.81 | 0.77 | 1 |  | | | | | | | | | | | | | |
| **ITEM34** | 0.64 | 0.72 | 0.68 | 0.79 | 0.66 | 0.77 | 0.80 | 0.80 | 0.86 | 0.81 | 1 |  | | | | | | | | | | | | |
| **ITEM36** | 0.73 | 0.70 | 0.76 | 0.79 | 0.75 | 0.81 | 0.82 | 0.81 | 0.88 | 0.87 | 0.89 | 1 |  | | | | | | | | | | | |
| **ITEM37** | 0.67 | 0.73 | 0.71 | 0.81 | 0.71 | 0.78 | 0.83 | 0.83 | 0.84 | 0.85 | 0.90 | 0.90 | 1 |  | | | | | | | | | | |
| **ITEM40** | 0.61 | 0.64 | 0.71 | 0.74 | 0.69 | 0.80 | 0.84 | 0.84 | 0.81 | 0.72 | 0.84 | 0.81 | 0.82 | 1 |  | | | | | | | | | |
| **ITEM42** | 0.63 | 0.66 | 0.72 | 0.75 | 0.69 | 0.81 | 0.83 | 0.81 | 0.84 | 0.75 | 0.85 | 0.86 | 0.79 | 0.87 | 1 |  | | | | | | | | |
| **ITEM46** | 0.68 | 0.66 | 0.73 | 0.77 | 0.68 | 0.83 | 0.81 | 0.78 | 0.82 | 0.76 | 0.88 | 0.87 | 0.83 | 0.85 | 0.89 | 1 |  | | | | | | | |
| **ITEM54** | 0.62 | 0.59 | 0.72 | 0.68 | 0.67 | 0.74 | 0.72 | 0.73 | 0.79 | 0.78 | 0.81 | 0.86 | 0.78 | 0.72 | 0.79 | 0.82 | 1 |  | | | | | | |
| **ITEM55** | 0.66 | 0.64 | 0.71 | 0.71 | 0.73 | 0.80 | 0.78 | 0.76 | 0.75 | 0.77 | 0.78 | 0.81 | 0.80 | 0.77 | 0.74 | 0.84 | 0.86 | 1 |  | | | | | |
| **ITEM59** | 0.77 | 0.72 | 0.81 | 0.76 | 0.78 | 0.84 | 0.92 | 0.83 | 0.87 | 0.86 | 0.84 | 0.89 | 0.87 | 0.84 | 0.86 | 0.87 | 0.84 | 0.87 | 1 |  | | | | |
| **ITEM61** | 0.61 | 0.69 | 0.68 | 0.81 | 0.70 | 0.83 | 0.78 | 0.79 | 0.82 | 0.75 | 0.85 | 0.86 | 0.87 | 0.83 | 0.84 | 0.89 | 0.78 | 0.75 | 0.84 | 1 |  | | | |
| **ITEM64** | 0.66 | 0.69 | 0.74 | 0.73 | 0.73 | 0.76 | 0.86 | 0.83 | 0.79 | 0.85 | 0.86 | 0.87 | 0.87 | 0.82 | 0.79 | 0.79 | 0.78 | 0.78 | 0.89 | 0.82 | 1 |  | | |
| **ITEM66** | 0.61 | 0.54 | 0.59 | 0.67 | 0.65 | 0.72 | 0.68 | 0.67 | 0.73 | 0.70 | 0.69 | 0.77 | 0.75 | 0.69 | 0.66 | 0.72 | 0.77 | 0.81 | 0.79 | 0.75 | 0.78 | 1 |  | |
| **ITEM69** | 0.60 | 0.67 | 0.75 | 0.78 | 0.70 | 0.79 | 0.79 | 0.76 | 0.75 | 0.79 | 0.81 | 0.81 | 0.86 | 0.80 | 0.74 | 0.84 | 0.77 | 0.80 | 0.84 | 0.83 | 0.83 | 0.71 | 1 |  |
| **ITEM72** | 0.71 | 0.72 | 0.77 | 0.79 | 0.75 | 0.87 | 0.85 | 0.83 | 0.84 | 0.80 | 0.84 | 0.90 | 0.84 | 0.83 | 0.85 | 0.87 | 0.83 | 0.82 | 0.89 | 0.89 | 0.84 | 0.78 | 0.80 | 1 |

Mean inter-item correlations = 0.77

**Online Resource 10. Comparison of design-based and two-level maximum model approaches (total sample)**

| **Item** | **λ_d_** | **δ_d_** | **λ_m_** | **δ_m_** | **ICC*** |
| --- | --- | --- | --- | --- | --- |
| **4** | 0.76 | 0.42 | 0.77 | 0.41 | 0.007 |
| **7** | 0.76 | 0.42 | 0.76 | 0.42 | 0.013 |
| **9** | 0.81 | 0.34 | 0.79 | 0.38 | 0.012 |
| **12** | 0.84 | 0.29 | 0.86 | 0.26 | 0.008 |
| **14** | 0.76 | 0.42 | 0.78 | 0.39 | 0.023 |
| **15** | 0.90 | 0.19 | 0.90 | 0.19 | 0.036 |
| **17** | 0.91 | 0.17 | 0.91 | 0.17 | 0.002 |
| **23** | 0.91 | 0.17 | 0.90 | 0.19 | 0.034 |
| **26** | 0.89 | 0.21 | 0.89 | 0.21 | 0.000 |
| **28** | 0.86 | 0.26 | 0.86 | 0.26 | 0.028 |
| **34** | 0.92 | 0.15 | 0.91 | 0.17 | 0.000 |
| **36** | 0.94 | 0.12 | 0.95 | 0.10 | 0.020 |
| **37** | 0.93 | 0.14 | 0.93 | 0.14 | 0.000 |
| **40** | 0.90 | 0.19 | 0.90 | 0.19 | 0.010 |
| **42** | 0.87 | 0.24 | 0.88 | 0.23 | 0.006 |
| **46** | 0.90 | 0.19 | 0.90 | 0.19 | 0.000 |
| **54** | 0.88 | 0.23 | 0.87 | 0.24 | 0.000 |
| **55** | 0.89 | 0.21 | 0.87 | 0.24 | 0.022 |
| **59** | 0.94 | 0.12 | 0.94 | 0.12 | 0.000 |
| **61** | 0.89 | 0.21 | 0.89 | 0.21 | 0.019 |
| **64** | 0.90 | 0.19 | 0.90 | 0.19 | 0.000 |
| **66** | 0.80 | 0.36 | 0.81 | 0.34 | 0.002 |
| **69** | 0.91 | 0.17 | 0.92 | 0.15 | 0.000 |
| **72** | 0.92 | 0.15 | 0.92 | 0.15 | 0.000 |

Number of clusters = 35. Average cluster size = 11.43. λ_d_ = within-level factor loading estimates for the design-based approach. δ_d_ = uniqueness terms for the design-based approach. λ_m_ = within-level factor loading estimates for the two-level maximum model approach. δ_m_ = uniqueness terms for the two-level maximum model approach. ICC = intraclass correlation coefficient. The Model fit for the design-based model was CFI = 0.994, TLI = 0.994, RMSEA = 0.053, SRMR = 0.030. The Model fit for the two-level maximum model was CFI = 0.997, TLI = 0.993, RMSEA = 0.025, SRMR (within) = 0.037, SRMR (between) = 0.017. *In terms of teachers (clusters), a total of n = 10 participants in the calibration sub-sample answered with “other teacher, not listed” and a total of n = 9 participants in the calibration sub-sample responded with “prefer not to say” to the name of the teacher question. These two types of responses were used to group participants into two different clusters for the analysis (all participants in the validation sample completed the name of the teacher being rated question). Discarding these participants in the analysis produced small variations on item ICC values, as follows (in order): 0.006, 0.010, 0.018, 0.010, 0.025, 0.025, 0.000, 0.025, 0.000, 0.024, 0.000, 0.023, 0.000, 0.006, 0.008, 0.001, 0.000, 0.021, 0.000, 0.001, 0.000, 0.004, 0.000, 0.000.

**Online Resource 11. Participant characteristics at pre-intervention in the new MBCT-TiF sample for an evaluation of convergent and discriminant validity**

| **Variables** | **MBCT-TiF (N=71)** |
| --- | --- |
| Age, mean (SD) | 51.59 (12.43) |
| Gender |  |
| Female, n (%) | 50 (70.40) |
| Male, n (%) | 20 (28.20) |
| Country |  |
| UK, n (%) | 50 (70.40) |
| Other, n (%) | 21 (29.60) |
| Occupation |  |
| Employed, n (%) | 51 (71.80) |
| Unemployed, n (%) | 3 (4.20) |
| Student, n (%) | 5 (7.00) |
| Retired, n (%) | 9 (12.70) |
| Previous mindfulness course |  |
| MBCT, n (%) | 52 (73.20) |
| MBSR, n (%) | 19 (26.80) |
| Years since previous course, mean (SD) | 3.85 (3.53) |
| Well-being*, M (SD) | 46.13 (7.73) |

M = mean. SD = standard deviation. Md = median. IQR = interquartile range. n = frequencies. % = percentages. *Well-being was measured using the Warwick-Edinburgh Mental Well-being Scale (WEMWBS).

**Online Resource 12. Fit indices and internal consistency for the measurement models using confirmatory factor analysis in the MBCT-TiF sub-sample.**

|  | **CFI** | **TLI** | **RMSEA** | **SRMR** | **ω** |
| --- | --- | --- | --- | --- | --- |
| **MBI:PAT** | 0.99 | 0.99 | 0.05 (0.02, 0.07) | 0.06 | 0.99 |
| **Expectations** (pre-) | 1.00 | 1.00 | 0.02 (0.00, 0.04) | 0.03 | 0.92 |
| **Credibility** (post-) | 1.90 | 1.00 | 0.06 (0.03, 0.09) | 0.03 | 0.96 |
| **WEMWBS** (pre-) | 0.99 | 0.99 | 0.07 (0.04, 0.10) | 0.06 | 0.95 |
| **WEMWBS2** (post-) | 0.99 | 0.99 | 0.08 (0.04, 0.11) | 0.05 | 0.97 |

MBI:PAT = Participants’ perspective on the quality of mindfulness-based intervention teaching. WEMWBS = Warwick-Edinburgh Mental Well-being Scale. CFI = comparative fit index. TLI = Tucker–Lewis index. RMSEA = root-mean-square error of approximation. SRMR = standardised root mean square residual. ω = McDonald’s omega. Overall health and potential harm were not evaluated since they consist of single-item measures.

**Online Resource 13. Convergent and discriminant validity of the MBI:PAT: raw relationships (MBCT-TiF sub-sample)**

|  | **r** | **95% CI** | **p** | **CFI** | **TLI** | **RMSEA** | **90% CI** | **SRMR** |
| --- | --- | --- | --- | --- | --- | --- | --- | --- |
| **Expectations** (pre-) | 0.12 | 0.02, 0.23 | 0.02 | 0.99 | 0.99 | 0.04 | 0.01, 0.06 | 0.06 |
| **Credibility** (post-) | 0.69 | 0.59, 0.78 | <0.001 | 0.99 | 0.99 | 0.06 | 0.04, 0.07 | 0.07 |
| **Mental well-being** (pre-) | 0.04 | -0.10, 0.19 | 0.56 | 0.98 | 0.98 | 0.05 | 0.04, 0.06 | 0.10 |
| **Mental well-being** (post-) | 0.22 | 0.10, 0.35 | <0.001 | 0.99 | 0.99 | 0.04 | 0.01, 0.05 | 0.08 |
| **Overall health** (pre-) | 0.02 | -0.07, 0.12 | 0.62 | 0.99 | 0.99 | 0.04 | 0.01, 0.06 | 0.06 |
| **Potential harm** (post-) | -0.39 | -0.55, -0.24 | <0.001 | 0.99 | 0.99 | 0.05 | 0.01, 0.07 | 0.06 |

WEMWBS = Warwick-Edinburgh Mental Well-being Scale. r = standardised correlation. 95% CI = 95% confidence interval. CFI = comparative fit index. TLI = Tucker–Lewis index. RMSEA = root-mean-square error of approximation. SRMR = standardised root mean square residual.
