# Supplementary material for: Participants’ Perspective on the Competence of Mindfulness-Based Interventions Teaching: Development and Validation of the Mindfulness-Based Interventions-Participants’ Assessment of Teaching (MBI:PAT) Questionnaire
Source: Mindfulness (N Y). 2025 Sep 3;16(10):2825–44. doi: 10.1007/s12671-025-02668-8 (PMC12660462; doi:10.1007/s12671-025-02668-8)
Supplement: Supplementary file 2 — Supplementary file2 (DOCX 21.1 KB) [file 12671_2025_2668_MOESM2_ESM.docx]

**Appendix A**

**Mindfulness-Based Interventions-Participants’ Assessment of Teaching (MBI:PAT)**

**We are interested in your impressions of the teaching you received during the mindfulness course you completed. Please rate each of the following statements according to the scale provided. When choosing your ratings, consider the whole mindfulness course.**

| **Statements** | **Not at all** | **A bit** | **Somewhat** | **Mostly good** | **Very much so, excellent** | **Outstanding!** |
| --- | --- | --- | --- | --- | --- | --- |
| 1. The classes flowed well together, with each lesson topic feeling as if it followed on from the last. | 1 | 2 | 3 | 4 | 5 | 6 |
| 2. The teacher responded to the learning needs of the class (for example, spending more time on something to help people understand). | 1 | 2 | 3 | 4 | 5 | 6 |
| 3. The teaching was pitched at the right level for me. | 1 | 2 | 3 | 4 | 5 | 6 |
| 4. The teacher was organised. | 1 | 2 | 3 | 4 | 5 | 6 |
| 5. The pace of the classes was right for me (for example, activities were not too rushed or too slow). | 1 | 2 | 3 | 4 | 5 | 6 |
| 6. The teacher appeared to be themselves (genuine/authentic) during the classes. | 1 | 2 | 3 | 4 | 5 | 6 |
| 7. I felt listened to by my teacher. | 1 | 2 | 3 | 4 | 5 | 6 |
| 8. I felt able to speak openly to the teacher. | 1 | 2 | 3 | 4 | 5 | 6 |
| 9. The teacher seemed genuinely interested in participants’ experiences during practices. | 1 | 2 | 3 | 4 | 5 | 6 |
| 10. I felt involved in the classes. | 1 | 2 | 3 | 4 | 5 | 6 |
| 11. The teacher appeared to be mindful whilst teaching the classes. | 1 | 2 | 3 | 4 | 5 | 6 |
| 12. The teacher responded to the needs of the group in the moment. | 1 | 2 | 3 | 4 | 5 | 6 |
| 13. The teacher responded calmly to challenging situations. | 1 | 2 | 3 | 4 | 5 | 6 |
| 14. I felt the teacher was accepting and non-judgmental. | 1 | 2 | 3 | 4 | 5 | 6 |
| 15. I got the impression that the teacher followed what they taught in class in their everyday life. | 1 | 2 | 3 | 4 | 5 | 6 |
| 16. When guiding mindfulness practices, the teacher’s instructions helped me to understand what to do when my mind wandered. | 1 | 2 | 3 | 4 | 5 | 6 |
| 17. The teacher helped me understand how awareness of thoughts, feelings and body sensations can be helpful. | 1 | 2 | 3 | 4 | 5 | 6 |
| 18. The teacher helped me learn from my experiences during mindfulness practices. | 1 | 2 | 3 | 4 | 5 | 6 |
| 19. The teacher was effective at supporting my learning. | 1 | 2 | 3 | 4 | 5 | 6 |
| 20. The teacher seemed confident when teaching. | 1 | 2 | 3 | 4 | 5 | 6 |
| 21. The teacher helped me to feel included in the group. | 1 | 2 | 3 | 4 | 5 | 6 |
| 22. The teacher dealt skilfully with challenging group members (for example, people who argued with the teacher or those who talked too much). | 1 | 2 | 3 | 4 | 5 | 6 |
| 23. The teacher supported the group well during difficult moments. | 1 | 2 | 3 | 4 | 5 | 6 |
| 24. The teacher's style of leading seemed right for the group. | 1 | 2 | 3 | 4 | 5 | 6 |
